# Hybrid Models Identified a 12-Gene Signature for Lung Cancer Prognosis and Chemoresponse Prediction

**DOI:** 10.1371/journal.pone.0012222

**Published:** 2010-08-17

**Authors:** Ying-Wooi Wan, Ebrahim Sabbagh, Rebecca Raese, Yong Qian, Dajie Luo, James Denvir, Val Vallyathan, Vincent Castranova, Nancy Lan Guo

**Affiliations:** 1 Mary Babb Randolph Cancer Center, West Virginia University, Morgantown, West Virginia, United States of America; 2 The Pathology and Physiology Research Branch, Health Effects Laboratory Division, National Institute for Occupational Safety and Health, Morgantown, West Virginia, United States of America; Deutsches Krebsforschungszentrum, Germany

## Abstract

**Background:**

Lung cancer remains the leading cause of cancer-related deaths worldwide. The recurrence rate ranges from 35–50% among early stage non-small cell lung cancer patients. To date, there is no fully-validated and clinically applied prognostic gene signature for personalized treatment.

**Methodology/Principal Findings:**

From genome-wide mRNA expression profiles generated on 256 lung adenocarcinoma patients, a 12-gene signature was identified using combinatorial gene selection methods, and a risk score algorithm was developed with *Naïve Bayes*. The 12-gene model generates significant patient stratification in the training cohort HLM & UM (*n* = 256; log-rank *P* = 6.96e-7) and two independent validation sets, MSK (*n* = 104; log-rank *P* = 9.88e-4) and DFCI (*n* = 82; log-rank *P* = 2.57e-4), using Kaplan-Meier analyses. This gene signature also stratifies stage I and IB lung adenocarcinoma patients into two distinct survival groups (log-rank *P*<0.04). The 12-gene risk score is more significant (hazard ratio = 4.19, 95% CI: [2.08, 8.46]) than other commonly used clinical factors except tumor stage (III vs. I) in multivariate Cox analyses. The 12-gene model is more accurate than previously published lung cancer gene signatures on the same datasets. Furthermore, this signature accurately predicts chemoresistance/chemosensitivity to Cisplatin, Carboplatin, Paclitaxel, Etoposide, Erlotinib, and Gefitinib in NCI-60 cancer cell lines (*P*<0.017). The identified 12 genes exhibit curated interactions with major lung cancer signaling hallmarks in functional pathway analysis. The expression patterns of the signature genes have been confirmed in RT-PCR analyses of independent tumor samples.

**Conclusions/Significance:**

The results demonstrate the clinical utility of the identified gene signature in prognostic categorization. With this 12-gene risk score algorithm, early stage patients at high risk for tumor recurrence could be identified for adjuvant chemotherapy; whereas stage I and II patients at low risk could be spared the toxic side effects of chemotherapeutic drugs.

## Introduction

Lung cancer is the leading cause of cancer-related deaths in industrialized countries [1]. Local and distant recurrence is the major cause of treatment failure (i.e. deaths) in lung cancer. Currently, surgery is the foremost treatment option for patients with stage I non-small cell lung cancer (NSCLC). However, 35–50% of stage I NSCLC patients will relapse within 5 years [2], [3]. It remains a critical challenge to determine the risk for recurrence in early-stage cancer patients. Patients at high risk for recurrence might benefit from adjuvant chemotherapy, whereas those with a low risk for tumor recurrence might be spared of the side effects of chemotherapy. Following this, another critical issue in clinics is to determine an individual patient's predisposition to a specific anticancer drug. The emerging use of biomarkers may enable physicians to make treatment decisions based on the specific characteristics of individual patients and their tumor, instead merely of on population statistics [4].

The advances in microarray technologies yield promise in the molecular prediction of individual clinical outcome. Such success is manifested by the commercial gene tests for breast cancer, Oncotype DX [5] and MammaPrint [6], [7]. Nevertheless, the high dimensionality of the data has complicated major diagnostic and prognostic breakthroughs [8] and puts a premium on innovative data mining methods. In current biomarker identification studies, genes are ranked according to their association with the clinical outcome, and the top ranked genes are included in the classifier. It has been noted that individual biomarkers showing strong association with the outcome are not necessarily good classifiers [9]–[11]. Furthermore, each individual gene selection algorithm has different strengths and limitations. A hybrid model combining multiple gene selection methods could better identify novel biomarkers from high-throughput data for clinical utility.

There have been a few studies on lung cancer prognosis by transcriptional profiling [12]–[19]. To date, there is no fully-validated and clinically applied model for predicting lung cancer recurrence [20]. This study presents a combinatorial gene selection system for the identification of a 12-gene lung cancer prognostic signature. This 12-gene signature is more accurate compared with previously published signatures in a multi-institutional study of lung adenocarcinoma (*n* = 442) [19]. This 12-gene signature could identify stage I and stage II patients who might benefit from adjuvant chemotherapy and who could be spared of it. Quantitative RT-PCR analyses of independent NSCLC tissue samples confirmed the gene expression patterns of the identified biomarkers in terms of tumor characteristics. A functional pathway analysis then revealed that the signature genes had interactions with well established cancer hallmarks, indicating the important roles of the signature genes in tumor initiation and progression. Furthermore, the 12-gene signature accurately predicted chemoresistance and chemosensitivity to Cisplatin, Carboplatin, Paclitaxel (Taxol), Etoposide, Gefitinib and Erlotinib in a panel of 60 cancer cell lines (NCI-60).

## Results

### Prognostic model system

In the post-genomic era, hybrid models that take advantage of different algorithms in different stages of gene selection are needed for biomarker discovery and disease classification. In this study, we combined statistical methods and machine learning algorithms to identify prognostic biomarkers of lung adenocarcinoma. The surgical resections collected from the University of Michigan Cancer Center (UM) and Moffitt Cancer Center (HLM) form the training set (*n* = 256), whereas the samples obtained from Memorial Sloan-Kettering Cancer Center (MSK, *n* = 104) and the Dana-Farber Cancer Institute (DFCI, *n* = 82) constitute two independent validation sets. The clinical characteristics of the patient cohorts were described in the previous publication [19].

**Figure 1:**
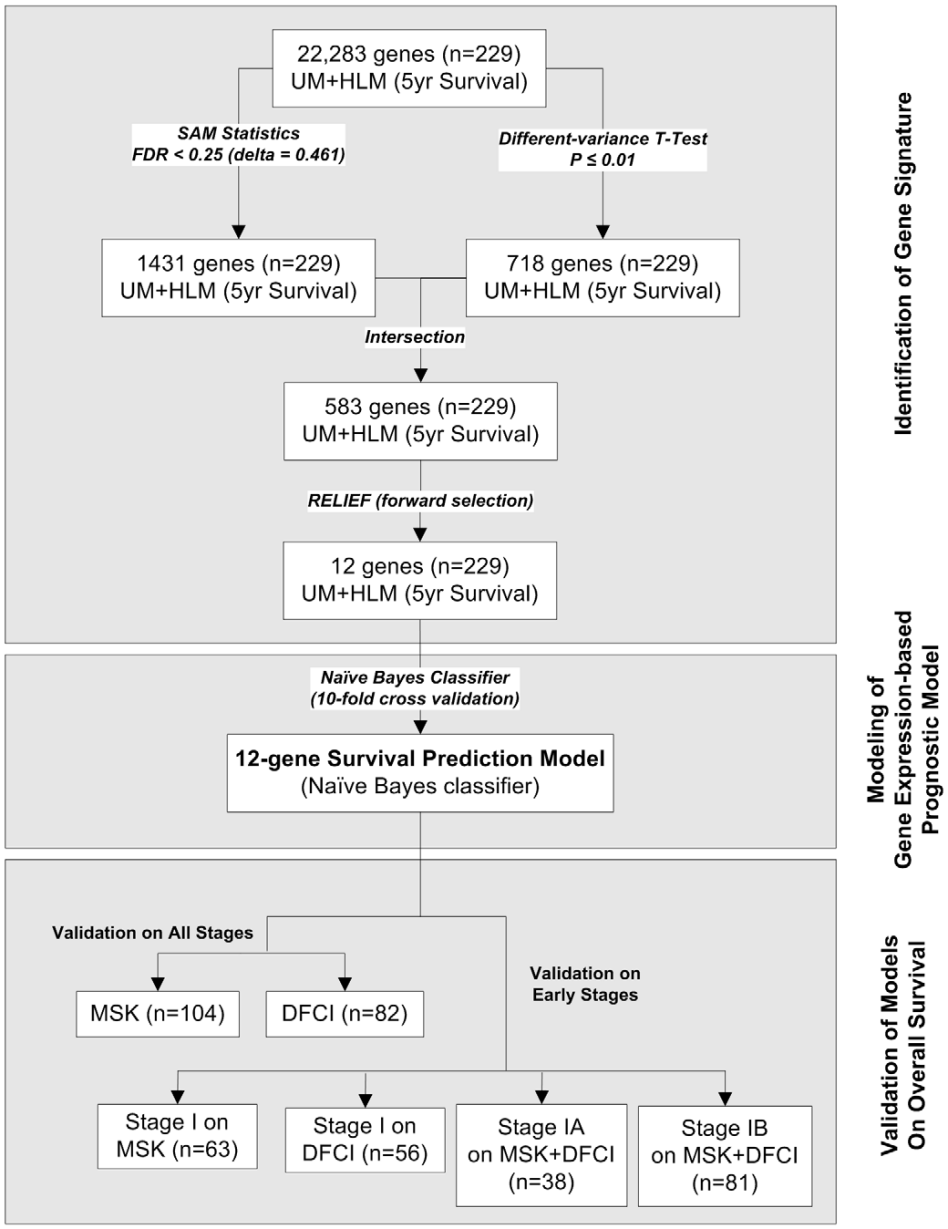
Overview of the study design for the identification of the 12-gene signature with combinatorial gene selection scheme and the construction of the expression-defined prognostic model.

The prognostic study includes three phases (Fig. 1): 1) identification of a small set of signature genes by combining Significance Analysis of Microarrays (SAM) [21], different-variance *t*-tests, and *Relief* algorithm from genome-scale transcriptional profiles of the training cohort (UM & HLM), 2) construction of a classifier using *Naive Bayes* algorithm to predict overall survival in lung cancer patients, and 3) validation of the gene expression-based prognostic model in two independent patient cohorts (MSK and DFCI). Independent test sets were used in the model validation and evaluation of the identified gene signature over previously published lung cancer prognostic signatures.

### Identification of a 12-gene prognostic signature

A combinatorial scheme with multiple gene selection methods was adopted in the process of identifying a lung cancer prognostic gene signature. The first step selected candidate genes from 22,283 probes quantified on the training cohort (*n* = 256). A combination of *t*-tests and SAM was then used to select genes with expression levels significantly different between low-risk (patient who survived longer than 5 years) and high-risk (those who died within 5 years following surgery) groups with a predefined false discovery rate. Twenty-seven censored cases with follow-up time less than 5 years were removed from this analysis due to the uncertainty of patient post-operative status. Specifically, a different-variance *t*-test selected 718 genes with significant differential expression (*P*<0.01) between the two prognosis groups. To control false discovery rate (*FDR*), SAM was used to select 1,431 genes that significantly differentiated the two prognostic groups at a *FDR* of 25% (*delta* = 0.46). There were 583 genes selected by both *t*-tests and SAM, and these were considered for the next stage of the analysis.

In order to refine the gene set into a more feasible size for clinical application, *Relief* algorithm implemented in WEKA 3.4 was used to rank each of these 583 genes in terms of the power to separate low-risk and high-risk groups. This ranked list was used in a step-wise forward selection to identify a gene subset with the highest prognostication accuracy. Specifically, starting from the top ranked gene, one gene was added at each step to the gene set, until the classification accuracy could not be improved by adding one more gene. This gene set was used to classify good-prognosis and poor-prognosis groups with *Naïve Bayes* algorithm. This process stopped when the addition of a new gene did not increase the classification accuracy as evaluated in a 10-fold cross validation. As a result, a 12-gene signature (Table 1) was identified which could provide the best prediction for overall survival.

**Table 1:** The identified 12-gene lung cancer prognostic signature.

Gene	Probe Set ID	Protein Functions	Classification
ATP6V0D1	212041_at	ATPase	Metabolism
PKLR	222078_at	Pyruvate kinase	Metabolism
SCLY	219808_at	Catalyzes the decomposition of L-selenocysteine to L-alanine and elemental selenium	Metabolism
SMPD1	209420_s_at	Converts sphingomyelin to ceramide	Metabolism
DLC1	210762_s_at	A candidate tumor suppressor gene	Oncogene
PDPK1	204524_at	Cell signal protein	Oncogene
ZAK	218833_at	Cell signal protein	Oncogene
STK24	208855_s_at	Protein kinase	Signaling Transduction
XPO1	208775_at	Mediates nuclear export of cellular proteins	Signaling Transduction
LMF1	46142_at	Maturation of specific proteins in the endoplasmic reticulum	Structure
FAM164A	205308_at	Unknown	N/A
CCDC99	221685_s_at	Cell cycle	Signaling Transduction

### Survival prediction using 12-gene prognostic model

Using mRNA expression profiles of the identified 12 genes as predictors, a prognostic classifier was constructed to stratify patients into low-risk (5-year survival) and high-risk (non-5-year survival) groups. The *Naïve Bayes* classifier implemented in WEKA 3.4 was used in the classification on UM & HLM training samples (low-risk *n* = 104; high-risk *n* = 125). Twenty-seven censored cases without sufficient follow-up information were removed in the model construction. Priors estimated by the model are 0.45 for low-risk class and 0.55 for high-risk class. Other parameters of the trained *Naïve Bayes* model, including the mean and standard deviation for each of the 12 genes in both low- and high-risk groups, are listed in Table 2.

**Table 2:** Parameters estimated in the 12-gene *Naïve Bayes* classifier.

Gene (attribute)	Low-risk mean ( 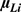 )	Low-risk standard deviation ( 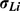 )	High-risk mean ( 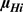 )	High-risk standard deviation ( 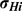 )
LMF1	101.6708	31.6461	88.6869	29.5986
DLC1	868.5886	578.3862	648.4284	530.6969
PKLR	14.3474	6.872	11.002	5.5501
ATP6V0D1	1388.054	398.6874	1209.6369	325.7233
CCDC99	277.1923	56.2284	300.0086	60.678
SCLY	58.3824	13.2988	63.6222	13.7703
PDPK1	297.6373	117.3514	253.7384	103.0455
FAM164A	264.8707	106.5128	223.8295	96.6066
SMPD1	278.5686	84.5316	239.3571	65.4393
XPO1	1674.3741	344.9824	1824.6274	400.4278
ZAK	132.694	67.7063	159.0546	79.1456
STK24	2248.6647	529.6098	2457.9982	576.496

**Figure 2:**
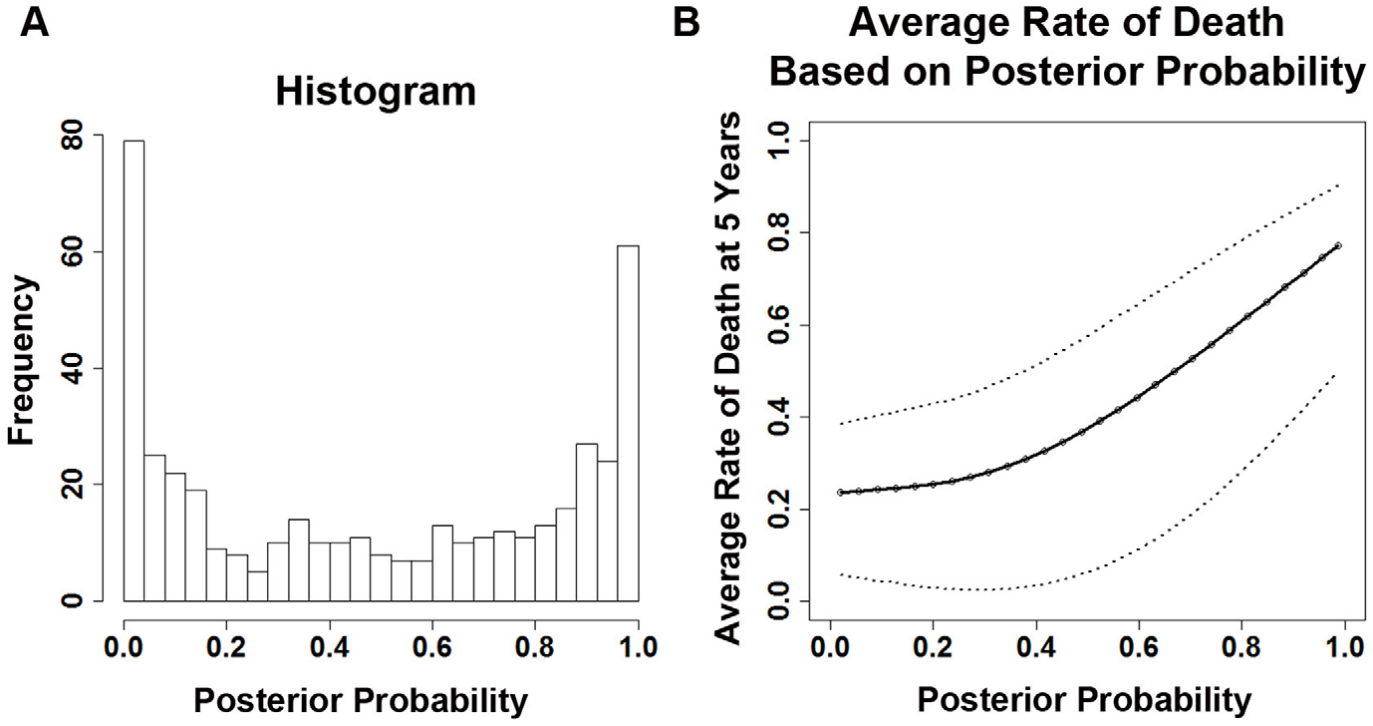
Association of the 12-gene risk score algorithm and lung cancer survival. (A). Histogram showing the distribution of the risk scores (posterior probabilities of high-risk) in 442 lung adenocarcinoma patients. (B). Average rate of death at five years after surgery corresponding to 12-gene risk score (posterior possibility). The dotted lines represent 95% confidence interval.

The *Naïve Bayes* classifier computes the posterior probability of death within 5 years after surgery in each patient. This posterior probability represents the risk for tumor recurrence in patients, since recurrence is the major cause of treatment failure (i.e. death) in lung cancer. Based on the posterior probability, a patient is classified into the high-risk group if the value is greater than 0.5; or into the low-risk group otherwise. The training model was evaluated in a 10-fold cross validation. Without parameter re-estimation, this model was then used to predict posterior probability representing the risk for tumor recurrence in each patient in two test sets (MSK and DFCI), as well as the censored cases left out of the model construction. The distribution of the posterior probability of 442 patients in this study was illustrated in Fig. 2A. After obtaining the predicted outcomes, Kaplan-Meier (KM) analysis was carried out to estimate the average survival probability at the 5-year mark following surgery. Results show that high-risk posteriors from the prognostic model are strongly associated with the 5-year survival probabilities (Fig. 2B). Patients with a high probability of tumor recurrence tend to be more likely to have treatment failure after surgery. This indicates that the high-risk posterior probability computed by the model is a good prognostic factor of lung cancer survival. The wide 95% confidence interval at posteriors ranging from 0.35 to 0.6 (Fig. 2B) might be due to the small sample size in this distribution (Fig. 2A). Furthermore, a posterior of 0.5 means that the chance of tumor recurrence is random, which also leads to a looser confidence interval.

**Figure 3:**
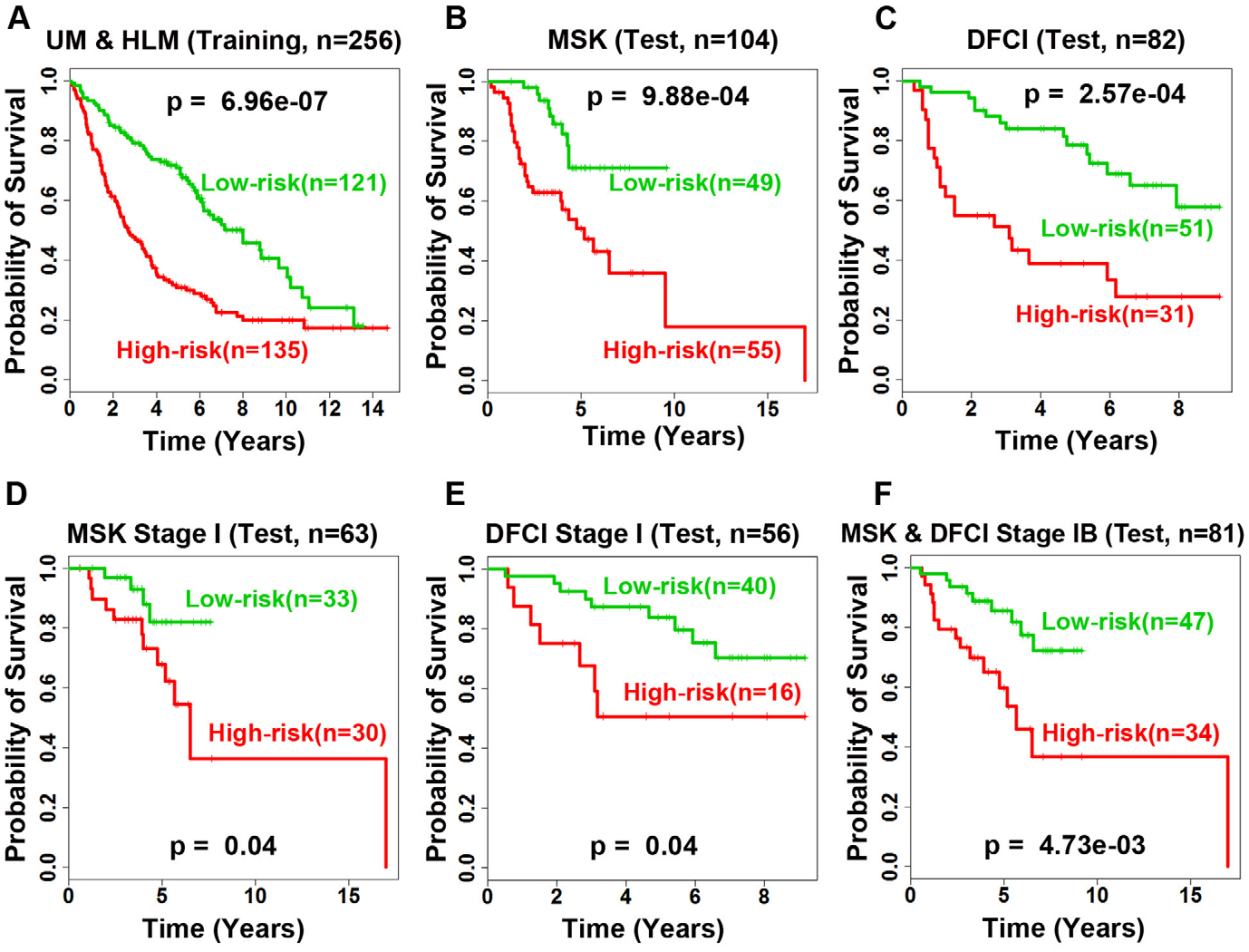
Kaplan-Meier analyses of the 12-gene prognostic model in patients with resectable lung adenocarcinoma. The model stratified patients into two significantly distinct (log-rank *P*<6.96e-7) prognostic groups in the training set (A) in 10-fold cross validation. The training model was applied to two test sets (B and C) and generated significant patient stratification. This model separated (log-rank *P*<0.04) stage I patients in both test sets (D and E) as well as stage IB patients (log-rank *P*<4.73e-3) from test sets (F).

Using the prognostic categorization scheme described above, the 12-gene signature separated patients into high- and low-risk groups with significantly distinct (log-rank *P* = 6.96e-7) post-operative survival on the training cohort in Kaplan-Meier analysis (Fig. 3A). This scheme generated significant patient stratification on independent validation sets MSK (log-rank *P* = 9.88e-4; Fig. 3B) and DFCI (log-rank *P* = 2.57e-4; Fig. 3C). The 3-year post-operative survival rate for low-risk groups is 79-94% in the studied cohorts, representing a significantly better prognosis compared with the corresponding high-risk groups for which the 3-year survival ranges from 48% to 63%. When 3-year survival was used to define high- and low-risk groups (high-risk: death within 3-y; low-risk: alive after 3-y), the 12-gene risk algorithm achieved a sensitivity (correctly predicted high-risk patients) of 73.65% in the training set, 86.96% in MSK, and 68.18% in DFCI, and a specificity (correctly predicted low-risk patients) of 59.21% in the training set, 57.75% in MSK, and 76.36% in DFCI (Table S9). The sensitivity and specificity of the 12-gene signature in predicting 5-year survival is also provided in Table S9.

In current practice, treatment for patients diagnosed with NSCLC is based on AJCC tumor stage. Surgical resection is the major treatment option for stage I NSCLC patients. However, about 35–50% of stage I NSCLC patients will develop and die from tumor recurrence within the five years following surgery [2], [3]. On the other hand, stage IB patients who received surgical resection followed by adjuvant chemotherapy showed improved survival rate [22]. Thus, we sought to explore whether the 12-gene signature could identify specific high-risk patients with stage I tumors. Results show that the 12-gene prognostic signature could reliably identify high-risk patients with stage I tumors on both the training cohort (results not shown) and two validation cohorts (log-rank *P* = 0.04; Fig. 3D and 3E). The prognostic model also separated high- and low-risk groups (log-rank P = 4.73e-3) within stage IB patients in the combined test sets (Fig. 3F). The 12-gene signature did not generate significant prognostic stratification on the stage IA patients (results not shown). These results demonstrate that the identified 12-gene signature is independent of and provides more refined prognosis than the current AJCC staging system. Using this model, stage I NSCLC patients could be advised to receive adjuvant chemotherapy according to the expression profiles of the 12 gene markers.

### Treatment selection for stage I and II NSCLC patients based on the 12-gene signature

**Figure 4:**
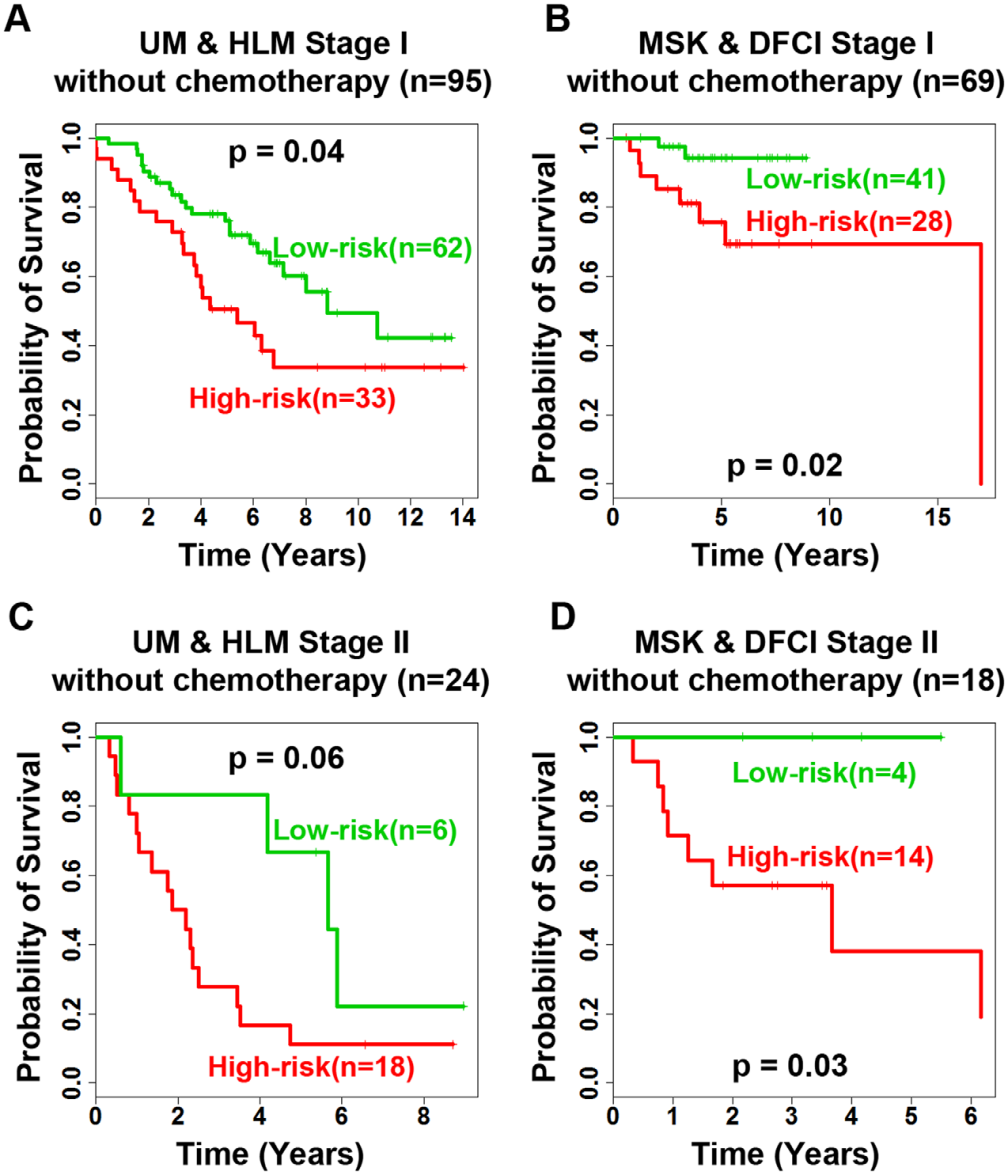
Evaluation of the 12-gene signature in treatment selection. The 12-gene signature separated high- and low-risk groups from patients who did not receive chemotherapy (log-rank *P*<0.05) in the following cohorts: (A) stage I patients from the training cohort, (B) stage I patients from two test cohorts, (C) stage II patients from the training cohort, (D) stage II patients from the two test cohorts.

In order to assess whether the 12-gene signature could be used for treatment selection for stage I and II non-small cell lung cancers, patients who did not receive chemotherapy were selected for further analysis. The prognostic model separated high- and low-risk stage I patients without chemotherapy in the training (UM & HLM; log-rank *P* = 0.04; Fig. 4A) and test cohorts (MSK & DFCI; log-rank *P* = 0.02; Fig. 4B). Similarly, the model differentiated high- and low-risk stage II patients without chemotherapy in the training (log-rank *P* = 0.06; Fig. 4C) and test cohorts (log-rank *P* = 0.03; Fig. 4D) in Kaplan-Meier analyses. The results indicate that this gene expression-defined prognostic model could reliably select patients with early stage NSCLC for adjuvant chemotherapy. Meanwhile, it could also spare some stage I and II NSCLC patients from chemotherapy based on the expression patterns of the identified gene markers in the tumors.

### Prognosis evaluation of the 12- gene signature with clinical covariates

To confirm the prognostic power of the identified 12-gene signature, the expression-defined prognostic model was evaluated with commonly used prognostic factors of lung cancer, including gender, age, and tumor stage on the combined testing cohorts (DFCI and MSK). The posterior probability of high-risk, termed as 12-gene risk score, was used as a covariate in the multivariate Cox analysis (Table 3). Without the 12-gene risk score, tumor stage was the only factor significantly (*P*<0.00006) associated with elevated risk of lung cancer death. When the 12-gene risk score was added to the multivariate Cox model, the 12-gene risk score demonstrated a strong association with the lung cancer survival (hazard ratio = 3.94, 95% CI: [2.07, 7.52]), and tumor stage remained significant (Table 3). Similarly, a comprehensive evaluation was carried out with all available clinical covariates and demographic data in the dataset, including smoking history, race, and tumor differentiation (Table 4). In this comprehensive evaluation, the 12-gene risk score remained a highly significant prognostic factor with a hazard ratio of 4.19 (95% CI: [2.08, 8.46]). In both multivariate analyses, the hazard ratios of the 12-gene risk score algorithm were higher than other clinical covariates except tumor stage (III vs. I), while there is no significant difference between the hazard ratio of the 12-gene signature and tumor stage. These results demonstrate that the 12-gene signature is a more accurate prognostic factor than some commonly used clinical parameters.

**Table 3:** Multivariate Cox proportional analysis of 12-gene risk score and major clinical covariates including gender, age, and tumor stage on testing cohorts (DFCI and MSK).

Variable*	*P*-value	Hazard Ratio (95% CI)ψ	
***Analysis without 12-gene risk score***			
Gender (Male)	0.22	1.34	(0.84–2.16)
Age at diagnosis (>60)	0.08	1.61	(0.95–2.74)
Tumor Stage			
Stage II	6.25E-05	2.91	(1.72–4.91)
Stage III	1.09E-05	4.16	(2.20–7.85)
***Analysis with 12-gene risk score***			
Gender (Male)	0.17	1.40	(0.87–2.26)
Age at diagnosis (>60)	0.29	1.34	(0.78–2.31)
Tumor Stage			
Stage II	3.47E-04	2.61	(1.54–4.43)
Stage III	7.40E-06	4.31	(2.28–8.16)
**12-gene risk score**	**3.10E-05**	**3.94**	**(2.07–7.52)**

*Gender was a binary variable (0 for female and 1 for male); age at diagnosis was a binary variable (0 for <60 years old and 1 otherwise); tumor stage was categorical variable of 3 categories (Stage I [as the reference group], Stage II, and Stage III).

ψ denotes confidence interval.

**Table 4:** Multivariate Cox proportional analysis of all available clinical covariates and 12-gene risk score on testing cohorts (DFCI and MSK).

Variable*	*P*-value	Hazard Ratio (95% CI)ψ	
***Analysis without 12-gene risk score***			
Gender (Male)	0.43	1.22	(0.74–1.99)
Age at diagnosis (>60)	0.05	1.70	(0.99–2.92)
Race			
Others/Unknown	0.28	0.43	(0.09–1.97)
White	0.10	0.28	(0.06–1.28)
Tumor differentiation			
Moderately differentiated	0.14	0.53	(0.23–1.24)
Poorly differentiated	0.70	1.17	(0.53–2.61)
Smoking history			
Smokers	0.62	0.84	(0.43–1.66)
Unknown	0.91	0.89	(0.11–7.10)
Tumor Stage	3.31E-04	2.72	(1.57–4.69)
Stage II	2.38E-05	4.93	(2.35–10.33)
Stage III	0.43	1.22	(0.74–1.99)
***Analysis with 12-gene risk score***			
Gender (Male)	0.38	1.25	(0.76–2.08)
Age at diagnosis (>60)	0.12	1.56	(0.89–2.72)
Race			
Others/Unknown	0.52	0.60	(0.13–2.77)
White	0.11	0.29	(0.07–1.32)
Tumor differentiation			
Moderately differentiated	0.17	0.56	(0.24–1.29)
Poorly differentiated	0.83	0.91	(0.41–2.06)
Smoking history			
Smokers	0.61	0.84	(0.43–1.64)
Unknown	0.79	0.75	(0.09–5.98)
Tumor Stage			
Stage II	1.37E-03	2.44	(1.41–4.22)
Stage III	5.12E-06	5.88	(2.75–12.58)
**12-gene risk score**	**6.34E-05**	**4.19**	**(2.08–8.46)**

*Gender was a binary variable (0 for female and 1 for male); age at diagnosis was a binary variable (0 for <60 years old and 1 otherwise); race was a categorical variable of 3 categories (African American [as the reference group], White, and Others [composed of Asian (5), Hawaiian or Pacific Islander (1), and unknown]); tumor grade was categorical variable of 3 categories (Well [as the reference group], Moderately, and Poorly differentiated); Smoking history was a categorical variable of 3 categories (Non-smokers, Smokers, and Unknown); tumor stage was categorical variable of 3 categories (Stage I [as the reference group], Stage II, and Stage III).

ψ denotes confidence interval.

### Comparison with other lung cancer gene signatures

**Figure 5:**
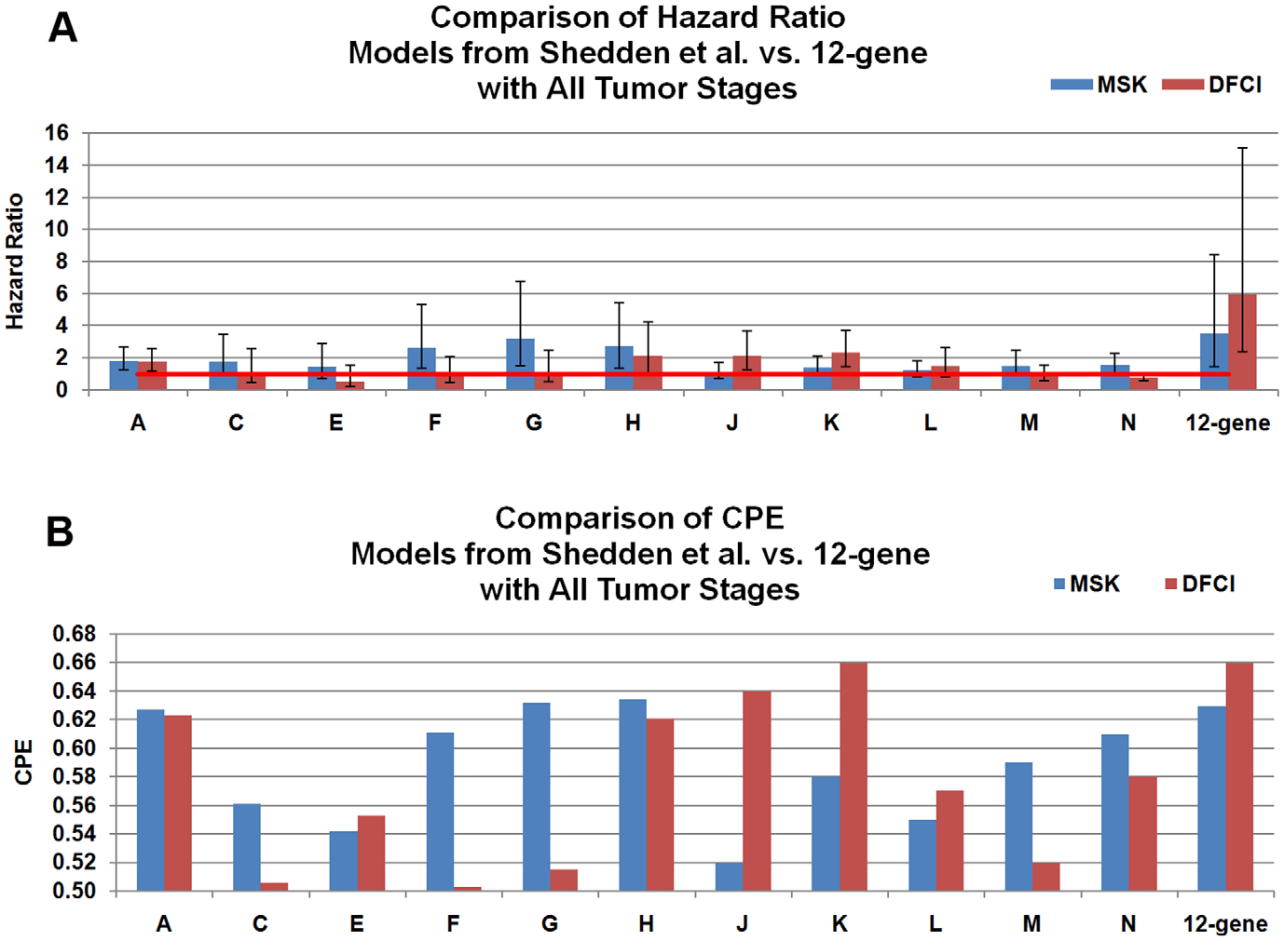
Comparison of the 12-gene risk score algorithm and various models presented by Shedden et al. [**19**] in two test sets in term of hazard ratios (A) and concordance probability estimate CPE (B).

In the study by Shedden et al. [19], prognostic classifiers were constructed with gene expression signatures alone or gene expression signatures combined with clinical covariates. Among twelve gene signatures identified in their study (Table S7), the best signature was reported as “method A” (referred to as “Shedden A” in this study), which contains about 9,591 genes/probes. In order to compare the predictive performance of our prognostic model with their best model, the estimated hazard ratio and the concordance probability estimate (CPE) of the models were evaluated. Hazard ratios greater than 1 indicate that patients with high predicted risk scores have poor clinical outcome. The model has strong predictive power if the CPE value is close to 1; CPE value close to 0.5 indicates that the model has poor predictive power (comparable to random prediction). Results show that the proposed 12-gene signature has the highest hazard ratio and CPE in both test sets when compared to the gene signatures from Shedden et al. [19] (Fig. 5).

To evaluate the 12-gene signature with previously published 14 lung cancer signatures [12], [13], [16]–[19], [22]–[25] (Table S6), Gene Set Enrichment Analysis (GSEA) was used to assess the enrichment of these signatures on 5-year survival. The normalized enrichment score (*NES*) and its corresponding false discovery rate (*FDR*) associated with each gene signature were evaluated on all 442 samples used in this study. In general, a gene set with high *NES* and low *FDR* is desired, as it indicates that the gene set expresses diversely with respect to the clinical outcome and the finding is unlikely to be by chance. In comparison to 14 other published gene signatures, the 12-gene signature exhibits high enrichment in patient group whose survival is longer than 5-year with significantly low *FDR* (absolute *NES* = 1.5; *FDR*<0.10) (Fig. S1). In this analysis, the most enriched signature with the lowest *FDR* was SHEDDEN_MH of 244 genes (absolute *NES* = 2.00; *FDR*<0.002). Overall, among the 15 gene sets studied, the 12-gene signature is one of the best lung cancer signatures evaluated with GSEA.

### RT-PCR Validation of gene expression patterns

**Figure 6:**
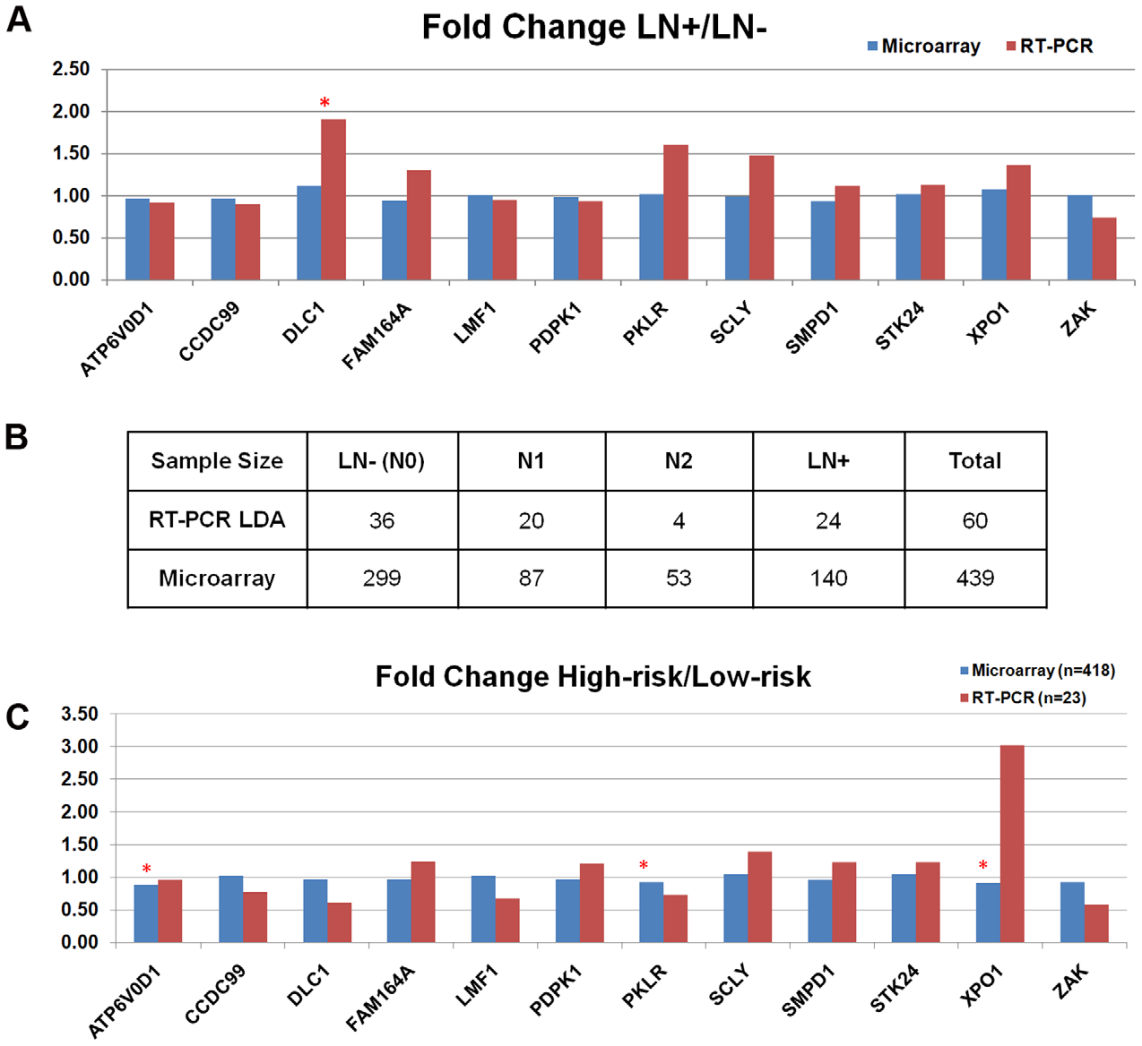
Comparison of expression patterns of 12 signature genes measured with DNA microarray and RT-PCR microfluidic low density arrays (LDA). Gene expression fold change in lymph node positive (LN+) patients vs. lymph node negative (LN−) patients was compared (A). Samples included in the fold change comparison were summarized in (B). Gene expression fold change in high-risk (death within 3-y) vs. low-risk (alive after 3-y) groups was also compared on patients with follow-up information (C). The RT-PCR data was normalized with POLR2A in a sample-wise manner. DNA microarray data were obtained from Shedden et al [19]. An asterisk (*) was put above a bar if that gene showed significant differential expression in *t*-tests (*P*<0.05).

In order to further confirm the expression patterns of the identified 12 genes, RT-PCR microfluidic low density arrays were used to analyze independent NSCLC tumor samples. First, the 12-gene expression patterns obtained from both microarray and RT-PCR were compared in terms of lymph node metastasis (Fig. 6A). On the RT-PCR data normalized with *POLR2A*, gene expression fold changes of the 12 genes in lymph node positive (LN+) versus lymph node negative (LN−) samples were compared with those in microarray data from Shedden et al [19]. The results show that the mRNA expression patterns of the 12-gene signature measured in both platforms are concordant in terms of lymph node metastasis. Then, to confirm the gene expression patterns in terms of overall survival, fold changes between low-risk (alive after 3 years) and high-risk (death within 3 years) groups were also compared (Fig. 6C). Three-year survival was used to keep the balance of the high-risk (*n* = 11) and low-risk (*n* = 12) groups in the RT-PCR cohort. All gene markers exhibited consistent expression patterns in over survival except *XPO1* in both platforms.

### Prediction of chemoresponse in NCI-60 cell lines

After substantiating the clinical relevance of the 12-gene signature in predicting lung adenocarcinoma overall survival, we sought to explore whether the signature can predict chemoresponse to anti-lung cancer agents, including Cisplatin, Carboplatin, Paclitaxel, Etoposide, Erlotinib, and Gefitinib. Here, the NCI-60 cell lines, regardless of tissue origin, were used in the study. For each drug, cancer cell lines that are either sensitive or resistant to the drug were included to build a chemoresponse classifier based on the 12-gene expression profiles in the cell lines. The performance of the classifiers was evaluated with leave-one-out cross validation (Table 5). Statistical significance of the classification was evaluated by comparing the overall accuracy of the 12-gene signature with that of 1000 random signatures of the same size using the same algorithm. The overall prediction accuracy of chemoresponse was 81% (*P*<0.004) for Paclitaxel (Taxol), 78% (*P*<0.001) for Carboplatin, 80% (*P*<0.005) for Cisplatin, 73% (*P*<0.017) for Etoposide, 79% (*P*<0.001) for Erlotinib, and 94% (*P*<0.001) for Gefitinib. These results demonstrate that the 12-gene signature accurately predicted sensitivity and resistance to common lung cancer chemotherapeutic agents in cancer cell lines.

**Table 5:** Prediction accuracy of chemoresponse in NCI-60 cell lines using the 12-gene signature.

Drug	Sensitivity (chemoresistance)	Specificity (chemosensitivity)	Overall accuracy	*P*-value*
**Carboplatin**	76% (19/25)	80% (16/20)	78% (35/45)	<0.001
**Paclitaxel**	72% (8/11)	87% (13/15)	81% (21/26)	0.004
**Cisplatin**	85% (22/26)	74% (14/19)	80% (36/45)	0.005
**Etoposide**	80% (16/20)	67% (14/21)	73% (30/41)	0.017
**Erlotinib**	79% (11/14)	80% (16/20)	79% (27/34)	0.001
**Gefitinib**	92% (11/12)	95% (20/21)	94% (31/33)	<0.001

*A *P*-value<0.05 represents that the overall accuracy of the 12-gene signature is significantly higher than that of random gene signatures with the same size using the same classifier in 1000 tests.

**Figure 7:**
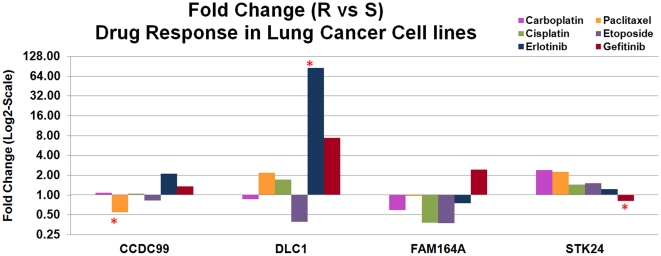
Genes with at least 1.5-fold expression fold change in resistant vs. sensitive lung cancer cell lines to four anticancer drugs. In the graph, differential expression with statistical significance (*P*<0.05, *t*-tests) is marked by a red asterisk.

The differential expression in sensitive and resistant lung cancer cell lines was analyzed for each signature gene. The drug responses of the lung cancer cell lines in the NCI-60 panel were provided in Table S8. Among the signature genes, the over-expression of *STK24* was linked to chemoresistance to all the studied drugs except Gefitinib in the lung cancer cell lines; whereas the over-expression of *FAM14A* was associated with chemosensitivity to all the studied drugs except Gefitinib in lung cancer cell lines. The under-expression of *STK24* was associated with resistance to Gefitinib (*P*<0.05). The under-expression of *CCDC99* was observed in resistance to Paclitaxel (*P*<0.05). The over-expression of *DLC1* was associated with chemoresistance to Erlotinib (*P*<0.05), Paclitaxel, and Cisplatin; whereas its under-expression was associated with chemoresistance to Etoposide and Carboplatin (not statistically significant) (Fig. 7).

*EGFR* mutation is a well known factor in drug response to Gefitinib and Erlotinib. In the NCI-60 cell lines, *EGFR* mutation was detected only in the SK-MEL-28 melanoma and RPMI-8226 myeloma lines, but not in any lung cancer cell lines [26]. We analyzed the raw expression levels of *EGFR* probe sets. Specifically, a fold change of 1.76 over-expression of *EGFR* (210984_x_at) was observed in Erlotinib resistant vs. sensitive lung cancer cell lines (*P*<0.05), whereas no significant differential expression of *EGFR* was observed in other studies drugs (results not shown). In the overall patient cohorts (*n* = 442) from Shedden et al [19], *EGFR* expression was not significantly associated with lung cancer overall survival in univariate Cox modeling.

### Functional pathway analysis

**Figure 8:**
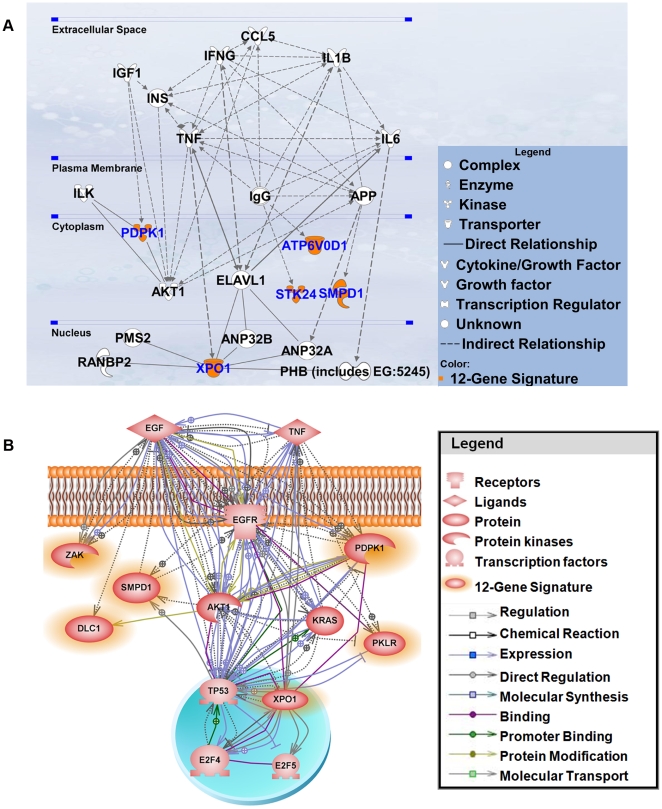
Functional pathway analysis of the 12 signature genes. (A) Using core analysis from Ingenuity Pathway Analysis (IPA), curated interactions were revealed among the identified signature genes and major lung cancer signaling pathways. (B) Six of the 12 signature genes also exhibited various curated interactions with eight prominent lung cancer hallmarks with Pathway Studio 7.0.

Having established the clinical relevance of the 12-gene prognostic signature, we sought to explore the functional involvement of this gene set in lung tumorigenesis and tumor progression. Two functional pathway analysis tools, Ingenuity Pathway Analysis (IPA) and Pathway Studio 7.0, were used to obtain curated molecular interactions related to the 12 genes. Results from IPA show that the signature genes interact with major cancer signaling pathways, such as *TNF* and *AKT* (Fig. 8A). Pathway Studio 7.0 was used to find interactions among the 12 genes and 13 major lung cancer hallmarks (*EGF*, *EGFR*, *KRAS*, *MET*, *RB1*, *TP53*, *E2F1*, *E2F2*, *E2F3*, *E2F4*, *E2F5*, *AKT1*, and *TNF*) reported in the literature. Pathway Studio revealed various types of interactions ranging from regulation to protein modification among the 12 genes and eight out of 13 cancer hallmarks (Fig. 8B). The functional pathway analysis suggests that the 12 signature genes are involved in lung cancer oncogenesis and tumor progression.

## Discussion

Lung cancer remains the leading cause of death worldwide. It is important to identify clinically relevant prognostic biomarkers to develop personalized treatment. More importantly, the discovered biomarkers may reveal fundamental molecular mechanisms of this deadly disease, and enhance our understanding of why patients with certain tumor molecular characteristics have a poor clinical outcome and how their outcome could be improved.

This study presents a hybrid model system for the identification of a 12-gene signature for lung cancer prognosis and chemoresponse prediction. The 12-gene signature accurately quantifies survival in patients with resectable lung adenocarcinoma, and provides significant prognostic categorization within stage I and IB patients, respectively. This signature reliably identified high-risk patients within stage I and II who did not receive chemotherapy. The gene expression-defined risk score is a more accurate prognostic factor than commonly used clinical parameters. This prognostic signature also predicts chemoresistance and chemosensitivity to several major anti-lung cancer drugs in NCI-60 cancer cell lines. Together, the results indicate that the 12-gene signature could be used to select early stage lung adenocarcinoma patients at high risk for tumor recurrence for adjuvant chemotherapy. Meanwhile, it may spare stage I and II low-risk patients from unnecessary chemotherapy. Furthermore, the 12-gene signature has the potential to be used to inform physicians which anticancer drugs should be used in treating a particular patient. The expression patterns of the 12-gene signature were confirmed in RT-PCR. Curated interactions between the signature genes and major cancer signaling hallmarks revealed in the functional pathway analysis provides further evidence that the 12-gene signature might be involved in lung cancer oncogenesis and tumor progression.

In the post-genomic era, innovative computational models are needed to identify clinically important disease markers. Given the current scale of high throughput data, a combinatorial gene selection scheme is needed at different stages of gene filtering. The choice to use a different feature selection technique depends on an evaluation with an independent classifier. If the classification performance cannot be further improved with the current algorithm, a different algorithm will be used to reduce the feature space. In this study, SAM and *t*-tests was used to identify candidate genes showing differential expression between two prognostic groups in the training set. SAM method is very similar to *t*-test. We used *t*-tests (*P*<0.01) to select genes with certain level of differential expression between two prognostic groups, and used SAM to control for false discovery rate (*FDR*<25%). The results from SAM and *t*-test are not exactly the same, because the SAM method adds a constant (*s*) in the denominator to ensure that genes with a very small variance in the samples and a small differential expression are not selected as significant markers. When *s* = 0, SAM is exactly the same as *t*-test [21]. In our study, genes that met both criteria (*P*<0.01 in *t*-tests and *FDR*<25% in SAM) were included for further analysis. From this candidate gene pool, *Relief* was then used to rank the importance of these genes in terms of prognostic classification for the selection of the final gene signature. This hybrid system was able to identify a small set of genes that are more accurate than previously published lung cancer gene signatures on the same datasets. We have experimented to change the threshold in SAM statistics. As a result, there were 87 genes with a *FDR*<10% and no genes were selected with a *FDR*<1% from the training set. The 87 genes were not able to generate significant stratification in all three patient cohorts. These results indicate that using SAM method alone is not sufficient to identify the most accurate prognostic gene signature. The hybrid models combining pooled variance *t*-tests and *Relief* algorithm also identified a 15-gene signature (Table S1). Among the two gene signatures, 16 genes (Table S2) share common biological functions (Table S5). The performance of these gene signatures (fitted as covariates in Cox model) is comparable to that of the 12-gene signature (Tables S3, S4 and S9; Fig. S2). The 15-gene signature was also validated with RT-PCR analysis of independent NSCLC tumor samples (Fig. S3). Overall, the hybrid models presented in this study are efficient and robust, and could be used in biomarker discovery in general.

## Materials and Methods

### Microarray profiles and patient samples

Gene expressions profiles analyzed in this study include 22,283 probes quantified with Affymetrix HG-U133A on 442 lung adenocarcinoma samples from Shedden et al [19]. This study cohort contains four data sets (University of Michigan, H. Lee Moffitt Cancer Center, Memorial Sloan-Kettering Cancer Center, and Dana-Farber Cancer Institute) contributed by six institutions. Tumors were collected by surgical resection from patients who have provided consent and protocols were approved by the Institutional Review Boards (IRB-Med) of the respective institutions. None of the patients received preoperative chemotherapy or radiation and least two years of follow-up information was available. Regions containing a minimum of 60% tumor cellularity were required for macrodissection, and in most instances tumor cellularity of at least 70–90% was identified for inclusion in the sample for RNA isolation. The raw microarray data are available from caArray website (https://array.nci.nih.gov/caarray/project/details.action?project.id=182).

A total of 91 NSCLC specimens to be used in RT-PCR analysis were obtained from West Virginia University Tissue Bank and the Cooperative Human Tissue Network (CHTN) (Ohio State University Tissue Bank, Columbus, OH). Tumor tissues were collected in surgical resections and were snap-frozen and stored at −80°C until used for RNA extraction. This study was approved with an IRB exemption from West Virginia University.

### RNA extraction, and quality and concentration assessments

Total RNA was extracted from snap-frozen tumor tissues using a RNeasy Fibrous Tissue Mini Kit according the manufacturer's protocol (Qiagen, USA). Total RNA was eluted in 30 µl of RNase-free water and stored at −80°C. The quality and integrity of the RNA was evaluated by visualizing the gel image and electropherogram for each sample using the 2100 Bioanalyzer (Agilent Technologies, CA). RNA assessed as having good quality from 74 tumor samples was included for further analysis. The RNA concentration of each sample was determined using a Nanodrop-1000 Spectrophotometer (NanoDrop Tech, Germany). Total RNA samples were analyzed on an Agilent 2100 Bioanalyzer RNA 6000 Nano LabChip.

### Generation of complementary DNA (cDNA)

The reverse transcriptase polymerase chain reaction was used to convert the high-quality single-stranded RNA samples to double-stranded cDNA, using an Applied Biosystems GeneAmp® PCR 9600 machine (Foster City, CA). For standardization across all samples, one microgram of RNA was used to generate cDNA.

### Real-time RT-PCR low-density arrays

Real-time RT-PCR assays of an independent patient cohort of NSCLC tumor samples were used to confirm the expression levels of the identified signature genes in microarray platform. The identified signature genes and three housekeeping genes were included in the experiment. The three housekeeping genes, *18S*, *UBC*, and *POLR2A*, were selected due to their confirmed constant mRNA expressions across samples [27].

We analyzed 74 tumor samples with high quality RNA using TaqMan microfluidic low-density array (LDA) plates on an ABI 7900HT Fast RT-PCR instrument (Applied Biosystems). The report was generated by the SDS2.3 software (Applied Biosystems). In the report, the number of cycles required to reach threshold fluorescence (Ct) and ΔC_T_ for each sample relative to the control gene defines the expression pattern for a gene. The gene expression data were further analyzed using the 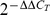 method [28].

### SAM statistics

Significance analysis of microarray (SAM) implemented in MultiExperiment Viewer (MeV; downloaded from http://www.tm4.org/mev.html) was used to identify statistically significant genes. The relative difference of the expression levels of each gene in two prognostic groups is computed and ranked (gene specific *t*-tests). Null distribution of the relative difference of each gene is generated by random permutations of patients' prognostic group labels. The expected relative difference of each gene is calculated as the mean of the relative differences of this gene in the null distribution. Genes with observed relative difference that varies from the expected relative difference by a certain threshold (*delta*) were identified as significant genes. For a specific *delta*, SAM provides a false discovery rate (*FDR*) based on the permutation analysis of expression data [21].

### Relief algorithm

Based on the genes selected with different variance *t*-tests and SAM statistics, *Relief* was used to rank these genes with WEKA 3.4. *Relief* evaluates the importance of a variable by repeatedly sampling an instance and checking the value of the given variable for the nearest instance from the same and different classes. The values of the attributes of the nearest neighbors are compared to the sampled instance and used to update the relevance scores for each attribute. As approximated in following equation, *Relief* computes the weight of attribute *A* as:
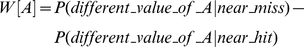

*Relief* assigns more weight to those attributes that have the same value for instances from the same class and differentiate between instances from different classes [29], [30].

### Naïve Bayes classifier

*Naïve Bayes* algorithm is based on Bayes theorem with the assumption that attributes are conditionally independent given the target class. A new sample with attribute values <*a_1_*, *a_2_*,*…*, *a_i_*> would be classified into the most probable class based on posterior probability and computed according to the Bayes theorem [31]. Specifically, the new sample would be classified into the class with the highest posterior probability, based on the following equation:
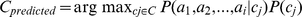
where *C* is the set containing all the classes for the problem and *c_j_* is a specific class.

Based on the conditional independence assumption, it holds true for the situation that given a class of instances, the probability of observing the conjunction of attributes *a_1_*, *a_2_*,*…*, *a_i_* would be the product of the probability of the individual attributes: 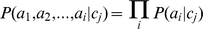 [31]. Therefore, a simpler form of the above equation used in *Naïve Bayes* classifier is as follows:
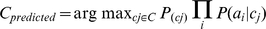



In this study, the *Naïve Bayes* algorithm was used to classify 5-year survival status in the training data with WEKA 3.4 [32]. Since gene expression is an attribute of continuous values, the probability density function for normal distribution was estimated to compute the probability of the gene in each class. Thus, the posterior probability for a new sample could be computed by:
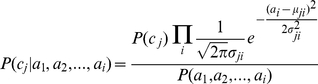
where 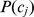 is the prior for class *j*, 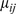 and 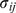 are estimated mean and standard deviation for the *i^th^* gene in class *j*.

### Functional Pathway Analysis

Ingenuity Pathway Analysis (Ingenuity Systems, Redwood City, CA), and Pathway Studio 7.0 (Ariadne Genomics, Rockville, MD) were used to derive curated molecular interactions, including both physical and functional interactions, and pathway relevance. The databases and software toolsets weigh and integrate information from numerous sources, including experimental repositories and text collections from published literature.

### Gene Set Enrichment Analysis (GSEA.)

GSEA (http://broad.harvard.edu/gsea/) was used to evaluate the enrichment of gene sets in prognostic groups defined by 5-year survival. All measured genes are ranked according to the differential expression between the two prognostic groups. An Enrichment Score (*ES*) for each gene set is computed by going through the ranked list from the top. *ES* is increased if the encountered gene is a member of the gene set or decreased if otherwise.

In this study, multiple gene sets were evaluated with the GSEA. In Multiple Hypothesis Testing, prognostic group labels are randomly permuted. A normalized enrichment score (*NES*) for each gene set is generated by averaging enrichment scores from all permutations. Statistical significance of the corresponding *NES* is indicated by false discovery rate (*FDR*) in the permutation analysis [33].

### Transcriptional Profiles in NCI-60 Cell Panel

Genome-wide mRNA expression profiles in NCI-60 cell lines [34] were retrieved with CellMiner (http://discover.nci.nih.gov/cellminer). The data were generated on Affymetrix U133A and normalized using the *GCRMA* method [35]. The signature genes were identified from the data file with gene symbols or UniGene Cluster IDs (for unknown genes).

### Drug activity profiles in NCI-60

The drug activity data in NCI-60 were retrieved from Developmental Therapeutic Program at NCI/NIH through DTP Data Search (http://dtp.nci.nih.gov/dtpstandard/dwindex/index.jsp). The latest screening results for each studied drug were used in the analysis. Growth inhibition was assessed from the changes in total cellular protein after 48 hours of drug treatment using a sulphorhodamine B assay. Drug activities (log_10_ GI_50_) were recorded across the 60 human cancer cell lines. GI_50_ is the concentration required to inhibit cell growth by 50% compared with untreated controls. The activity profile of an agent consists of 60 such activity values, one for each cell line.

### Defining Drug Sensitivity and Resistance

Drug activity data for Cisplatin, Carboplatin, Paclitaxel, and Etoposide was processed to define drug resistance and sensitivity in the NCI-60 lines as described before [36], [37]. Specifically, for each drug, log_10_ (GI_50_) values were normalized across the 60 cell lines. Cell lines with log_10_ (GI_50_) at least 0.5 SDs above the mean were defined as *resistant* to the drug. Those with log_10_ (GI_50_) at least 0.5 SDs below the mean were defined as *sensitive* to the drug. The remaining cell lines with log_10_ (GI_50_) within 0.5 SDs were defined as *intermediate* in the range of drug responses.

### Classification of chemosensitivity/resistance

The mRNA expression profiles of the 12-gene lung cancer signature were used to predict chemosensitivity/resistance in the cancer cell lines. For each drug, only *sensitive* and *resistant* cell lines were included in the analysis, while those with *intermediate* response were excluded from classification. A *k*-nearest neighbor method was used to classify chemoresponse to Paclitaxel. Neural network was used to classify drug response to Carboplatin, Gefitinib, and Erlotinib. Boosting trees (AdaBoost) was used to classify response to Etoposide. An ensemble learning method (Decorate) was used in classifying chemoresponse to Cisplatin. The classification results were evaluated with a leave-one-out cross validation. These algorithms were implemented in WEKA 3.4 [32].

### Differential expression analysis in resistant and sensitive lung cancer cell lines

Using the average expression values of each gene in the lung cancer cell lines in the NCI-60 panel, fold change of the gene expression in resistant cell lines versus sensitive cell lines was computed as follows:
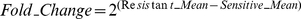
Where *Resistant_Mean* is the mean expression of the group of resistant cell lines and *Sensitive_Mean* is the mean expression of the group of sensitive cell lines. In this study, a value of 1.5 (1.5 for over-expressed and 0.67 for under-expressed) is the threshold used in deciding if a gene is expressing differently. Statistical significance of the fold change is computed using two-tail, unequal variance two-sample *t*-tests. It's considered statistically significant if a *p*-value is ≤0.05.

### Statistical Analysis

In Kaplan-Meier analysis, log-rank tests were used to assess the difference in probability of survival of different prognostic groups. Hazard ratio and concordance probability estimate (CPE) were used in the evaluation of different molecular prognostic signatures. If the model gives hazard ratio greater than 1, it means that patient samples predicted as high risk are more likely to have poor outcome. CPE values close to 1 represents high concurrence and good predictive power; CPE values close to 0.5 represents low concurrence and poor predictive power. All the analyses were performed with packages in *R* unless otherwise specified.

## Supporting Information

Table S1 A 15-gene lung cancer prognostic signature. This gene signature was identified using pooled-variance t-tests and RELIEF algorithm. The expression of the 15 genes were used as covariates in Cox model and median risk score from training set was used as the cutoff point.
(0.05 MB DOC)

Table S2 A 16-gene signature sharing common biological functions between 12- and 15-gene signatures (Table S5). Cox model was fitted with these 16 gene expression levels and 75th percentile of the risk scores from training set was used as the cutoff.
(0.05 MB DOC)

Table S3 Multivariate Cox proportional analysis of 15- and 16-gene risk score with major clinical covariates in lung cancer survival on testing cohorts (DFCI and MSK).
(0.05 MB DOC)

Table S4 Multivariate Cox proportional analysis of 15- and 16-gene risk score with all clinical covariates in lung cancer survival on testing cohorts (DFCI and MSK).
(0.08 MB DOC)

Table S5 Comparison of biological functions between 12-gene signature and 15-gene signature with curated database. The biological functions were obtained using Ingenuity Pathway Analysis (IPA).
(0.09 MB DOC)

Table S6 14 published lung cancer gene signatures evaluated in GSEA.
(0.05 MB DOC)

Table S7 Summary of gene selection and classification methods of molecular classifiers compared in Fig. 5. Gene signatures A-N were reported in (Shedden et al, 2008).
(0.05 MB DOC)

Table S8 Machine learning algorithm and genes used in chemoresponse prediction using 12-gene signature.
(0.05 MB DOC)

Table S9 Sensitivity and specificity of the 12-, 15- and 16-gene prognostic models.
(0.05 MB DOC)

Figure S1 Gene set enrichment analysis of the 12-gene signature along with 14 published gene signatures for NSCLC. A summary of the 14 gene signatures analyzed is listed in Table S6.
(0.10 MB TIF)

Figure S2 Evaluation of the 15-gene, 12-gene, and 16-gene prognostic models with molecular prognostic models presented by Shedden et al (2008). Hazard ratio (A, C) and concordance probability estimate (CPE) (B, D) were compared on patients in all stages (A, B) and stage I (C, D) of lung cancer. Error bars in (A) and (C) represent 95% confidence interval of hazard ratio.
(0.15 MB TIF)

Figure S3 Comparison of gene expression patterns of the 15-gene signature measured with DNA microarray and RT-PCR microfluidic low density arrays (LDA). Gene expression fold change in lymph node positive (LN+) patients vs. lymph node negative (LN−) patients was compared (A). Samples included in the fold change comparison are summarized in (B). On patient with follow-up information, gene expression fold change in high-risk patients vs. low-risk patients at 3-year period after surgery was also compared (C). The RT-PCR data were normalized with POLR2A in a sample-wise manner. DNA microarray data were obtained from Shedden et al (2008). Red asterisk (*) above the bar indicates the gene was differentially expressed t-test (P<0.05).
(0.22 MB TIF)
