# Supplementary material for: Hybrid Models Identified a 12-Gene Signature for Lung Cancer Prognosis and Chemoresponse Prediction
Source: PLoS One. 2010 Aug 17;5(8):e12222. doi: 10.1371/journal.pone.0012222 (PMC2923187; doi:10.1371/journal.pone.0012222)
Supplement: Table S1 — A 15-gene lung cancer prognostic signature. This gene signature was identified using pooled-variance t-tests and RELIEF algorithm. The expression of the 15 genes were used as covariates in Cox model and median risk score from training set was used as the cutoff point. (0.05 MB DOC) [file pone.0012222.s001.doc]

| **Probe Set ID** | **Gene** | **Functions** | **Classification** |
| --- | --- | --- | --- |
| 204854_at | GPR162 /// LEPREL2 | Collagen biosynthesis, folding, and assembly | Metabolism |
| 206150_at | CD27 | B-cell activation and immunoglobulin synthesis; signaling transduction | Oncogene |
| 205171_at | PTPN4 | Cell growth, differentiation, mitotic cycle, and oncogenic transformation | Oncogene |
| 201107_s_at | THBS1 | Cell-to-cell and cell-to-matrix interactions. | Oncogene |
| 210762_s_at | DLC1 | A candidate tumor suppressor gene | Oncogene |
| 218340_s_at | UBA6 | Ubiquitin-activating protein | Protein Degradation |
| 211327_x_at | HFE | Iron absorption | Signaling Transduction |
| 208772_at | ANKHD1 | Unknown | Structure |
| 211603_s_at | ETV4 | Cellular movement | Transcription |
| 207296_at | ZNF343 | Unknown | Transcription |
| 214717_at | DKFZp434H1419 | Unknown | N/A |
| 213779_at | EMID1 | Unknown | N/A |
| 215598_at | TTC12 | Binding | N/A |
| 201581_at | TXNDC13 | Cell redox homeostasis, electron transport chain | N/A |
| 205308_at | FAM164A | Unknown | N/A |
