# Supplementary material for: Hybrid Models Identified a 12-Gene Signature for Lung Cancer Prognosis and Chemoresponse Prediction
Source: PLoS One. 2010 Aug 17;5(8):e12222. doi: 10.1371/journal.pone.0012222 (PMC2923187; doi:10.1371/journal.pone.0012222)
Supplement: Table S2 — A 16-gene signature sharing common biological functions between 12- and 15-gene signatures (Table S5). Cox model was fitted with these 16 gene expression levels and 75th percentile of the risk scores from training set was used as the cutoff. (0.05 MB DOC) [file pone.0012222.s002.doc]

| **Probe Set ID** | **Gene** | **Functions** | **Classification** |
| --- | --- | --- | --- |
| 206150_at | CD27 | B-cell activation and immunoglobulin synthesis; signaling transduction | Oncogene |
| 205171_at | PTPN4 | Cell growth, differentiation, mitotic cycle, and oncogenic transformation | Oncogene |
| 201107_s_at | THBS1 | Cell-to-cell and cell-to-matrix interactions. | Oncogene |
| 211327_x_at | HFE | Iron absorption | Signaling Transduction |
| 211603_s_at | ETV4 | Cellular movement | Transcription |
| 201581_at | TXNDC13 | Cell redox homeostasis, electron transport chain | N/A |
| 212041_at | ATP6V0D1 | Atpase | Metabolism |
| 222078_at | PKLR | Pyruvate kinase | Metabolism |
| 219808_at | SCLY | Catalyzes the decomposition of L-selenocysteine to L-alanine and elemental selenium | Metabolism |
| 209420_s_at | SMPD1 | Converts sphingomyelin to ceramide | Metabolism |
| 210762_s_at | DLC1 | A candidate tumor suppressor gene | Oncogene |
| 204524_at | PDPK1 | Cell signal protein | Oncogene |
| 218833_at | ZAK | Cell signal protein | Oncogene |
| 208855_s_at | STK24 | Protein kinase | Signaling Transduction |
| 208775_at | XPO1 | Nuclear protein transport | Signaling Transduction |
| 46142_at | LMF1 | Maturation of specific proteins in the endoplasmic reticulum | Structure |
