# Supplementary material for: Hybrid Models Identified a 12-Gene Signature for Lung Cancer Prognosis and Chemoresponse Prediction
Source: PLoS One. 2010 Aug 17;5(8):e12222. doi: 10.1371/journal.pone.0012222 (PMC2923187; doi:10.1371/journal.pone.0012222)
Supplement: Table S3 — Multivariate Cox proportional analysis of 15- and 16-gene risk score with major clinical covariates in lung cancer survival on testing cohorts (DFCI and MSK). (0.05 MB DOC) [file pone.0012222.s003.doc]

| **Variable***** | **P value** | **Hazard Ratio (95% CI) ^ψ^** | |
| --- | --- | --- | --- |
| ***Analysis without risk score*** | | | |
| Gender (Male) | 0.22 | 1.34 | (0.84-2.16) |
| Age at diagnosis (>60) | 0.08 | 1.61 | (0.95-2.74) |
| Tumor Stage |  |  |  |
| Stage II | 6.25E-05 | 2.91 | (1.72-4.91) |
| Stage III | 1.09E-05 | 4.16 | (2.20-7.85) |
| ***Analysis with 15-gene risk score*** | | | |
| Gender (Male) | 0.20 | 1.36 | (0.85-2.18) |
| Age at diagnosis (>60) | 0.08 | 1.60 | (0.94-2.74) |
| Tumor Stage |  |  |  |
| Stage II | 1.32E-04 | 2.80 | (1.65-4.74) |
| Stage III | 4.82E-05 | 3.73 | (1.98-7.05) |
| **15-gene risk score** | **2.84E-04** | **1.99** | **(1.37-2.89)** |
| ***Analysis with 16-gene risk score*** | | | |
| Gender (Male) | 0.11 | 1.49 | (0.92-2.41) |
| Age at diagnosis (>60) | 0.18 | 1.44 | (0.84-2.48) |
| Tumor Stage |  |  |  |
| Stage II | 5.36E-05 | 2.97 | (1.75-5.03) |
| Stage III | 7.52E-07 | 5.19 | (2.70-9.96) |
| **16-gene risk score** | **6.24E-07** | **2.50** | **(1.33-3.59)** |

* Gender was binary variable (0 for female and 1 for male); age at diagnosis was a binary variable (0 for < 60 years old and 1 otherwise); tumor stage was categorical variable of 3 categories (Stage I [as the reference group], Stage II, and Stage III). Risk score was continuous variable, and the hazard ratio represents the relative risk between the mean risk scores of high- and low-risk groups.

**^ψ^** denotes confidence interval.
