# Supplementary material for: Hybrid Models Identified a 12-Gene Signature for Lung Cancer Prognosis and Chemoresponse Prediction
Source: PLoS One. 2010 Aug 17;5(8):e12222. doi: 10.1371/journal.pone.0012222 (PMC2923187; doi:10.1371/journal.pone.0012222)
Supplement: Table S4 — Multivariate Cox proportional analysis of 15- and 16-gene risk score with all clinical covariates in lung cancer survival on testing cohorts (DFCI and MSK). (0.08 MB DOC) [file pone.0012222.s004.doc]

| **Variable*** | **P value** | | **Hazard Ratio (95% CI)^ψ^** | |
| --- | --- | --- | --- | --- |
| ***Analysis without risk score*** | | | | |
| Gender (Male) | 0.43 | | 1.22 | (0.74-1.99) |
| Age at diagnosis (>60) | 0.05 | | 1.70 | (0.99-2.92) |
| Race |  | |  |  |
| Others/Unknown | 0.28 | | 0.43 | (0.09-1.97) |
| White | 0.10 | | 0.28 | (0.06-1.28) |
| Tumor Grade |  | |  |  |
| Moderately differentiated | 0.14 | | 0.53 | (0.23-1.24) |
| Poorly differentiated | 0.70 | | 1.17 | (0.53-2.61) |
| Smoking History |  | |  |  |
| Smokers | 0.62 | | 0.84 | (0.43-1.66) |
| Unknown | 0.91 | | 0.89 | (0.11-7.10) |
| Tumor Stage |  |  | |  |
| Stage II | 3.31E-04 | 2.72 | | (1.57-4.69) |
| Stage III | 2.38E-05 | 4.93 | | (2.35-10.33) |
| ***Analysis with 15-gene risk scores*** | | | | |
| Gender (Male) | 0.36 | | 1.26 | (0.77-2.06) |
| Age at diagnosis (>60) | 0.04 | | 1.75 | (1.02-3.01) |
| Race |  | |  |  |
| Others/ Unknown | 0.38 | | 0.50 | (0.11-2.31) |
| White | 0.14 | | 0.32 | (0.07-1.45) |
| Tumor differentiation |  | |  |  |
| Moderately differentiated | 0.16 | | 0.55 | (0.24-1.27) |
| Poorly differentiated | 0.99 | | 0.99 | (0.44-2.23) |
| Smoking History |  | |  |  |
| Smokers | 0.93 | | 0.97 | (0.49-1.91) |
| Unknown | 0.85 | | 1.22 | (0.15-9.89) |
| Tumor Stage |  |  | |  |
| Stage II | 2.61E-04 | 2.76 | | (1.60-4.77) |
| Stage III | 5.19E-05 | 4.66 | | (2.21-9.82) |
| **15-gene risk score** | **2.47E-03** | | **1.81** | **(1.23-2.65)** |
| ***Analysis with 16-gene risk score*** | | | | |
| Gender (Male) | 0.17 | | 1.42 | (0.86-2.35) |
| Age at diagnosis (>60) | 0.09 | | 1.63 | (0.93-2.85) |
| Race |  | |  |  |
| Others/ Unknown | 0.22 | | 0.38 | (0.08-1.77) |
| White | 0.05 | | 0.22 | (0.05-1.00) |
| Tumor differentiation |  | |  |  |
| Moderately differentiated | 0.16 | | 0.55 | (0.23-1.28) |
| Poorly differentiated | 0.96 | | 1.02 | (0.45-2.30) |
| Smoking History |  | |  |  |
| Smokers | 0.53 | | 0.81 | (0.41-1.59) |
| Unknown | 0.96 | | 0.94 | (0.12-7.54) |
| Tumor Stage |  |  | |  |
| Stage II | 2.37E-04 | 2.79 | | (1.62-4.83) |
| Stage III | 2.09E-06 | 6.34 | | (2.96-13.58) |
| **16-gene risk score** | **7.49E-07** | | **2.45** | **(1.72-3.50)** |

* Gender was binary variable (0 for female and 1 for male); age ge at diagnosis was a binary variable (0 for < 60 years old and 1 otherwise); race was a categorical variable of 3 categories (African American [as the reference group], White, and Others [composed of Asian (5) , Hawaiian or Pacific Islander (1), and unknown]); tumor grade was categorical variable of 3 categories (Well [as the reference group], Moderately, and Poorly differentiate); Smoking history was a categorical variable of 3 categories (Non-smokers, Smokers, and Unknown); tumor stage was categorical variable of 3 categories (Stage I [as the reference group], Stage II, and Stage III). Risk score was continuous variable, and the hazard ratio represents the relative risk between the mean risk scores of high- and low-risk groups.

**^ψ^** denotes confidence interval.
