# Supplementary material for: Hybrid Models Identified a 12-Gene Signature for Lung Cancer Prognosis and Chemoresponse Prediction
Source: PLoS One. 2010 Aug 17;5(8):e12222. doi: 10.1371/journal.pone.0012222 (PMC2923187; doi:10.1371/journal.pone.0012222)
Supplement: Table S5 — Comparison of biological functions between 12-gene signature and 15-gene signature with curated database. The biological functions were obtained using Ingenuity Pathway Analysis (IPA). (0.09 MB DOC) [file pone.0012222.s005.doc]

| **Category** | **Category** | **12-gene** | **15-gene** | **Common** |
| --- | --- | --- | --- | --- |
| **Diseases and Disorders** | Cancer |  |  | ✓ |
|  | Cardiovascular Disease |  | ✓ |  |
|  | Connective Tissue Disorders |  | ✓ |  |
|  | Dermatological Diseases and Conditions |  | ✓ |  |
|  | Genetic Disorder |  |  | ✓ |
|  | Hematological Disease |  |  | ✓ |
|  | Hepatic System Disease |  |  | ✓ |
|  | Immunological Disease |  |  | ✓ |
|  | Infection Mechanism | ✓ |  |  |
|  | Inflammatory Disease |  | ✓ |  |
|  | Inflammatory Response |  | ✓ |  |
|  | Metabolic Disease |  |  | ✓ |
|  | Neurological Disease |  |  | ✓ |
|  | Reproductive System Disease |  |  | ✓ |
|  | Respiratory Disease |  |  | ✓ |
|  | Skeletal and Muscular Disorders |  | ✓ |  |
| **Molecular and Cellular Functions** | Amino Acid Metabolism |  |  | ✓ |
|  | Antigen Presentation |  | ✓ |  |
|  | Carbohydrate Metabolism |  | ✓ |  |
|  | Cell Cycle |  | ✓ |  |
|  | Cell Death |  |  | ✓ |
|  | Cell Morphology |  | ✓ |  |
|  | Cell Signaling |  |  | ✓ |
|  | Cell-To-Cell Signaling and Interaction |  | ✓ |  |
|  | Cellular Assembly and Organization |  |  | ✓ |
|  | Cellular Compromise |  | ✓ |  |
|  | Cellular Development |  |  | ✓ |
|  | Cellular Function and Maintenance |  |  | ✓ |
|  | Cellular Growth and Proliferation |  |  | ✓ |
|  | Cellular Movement |  |  | ✓ |
|  | DNA Replication, Recombination, and Repair | ✓ |  |  |
|  | Drug Metabolism |  | ✓ |  |
|  | Gene Expression | ✓ |  |  |
|  | Lipid Metabolism |  |  | ✓ |
|  | Molecular Transport |  |  | ✓ |
|  | Nucleic Acid Metabolism |  | ✓ |  |
|  | Post-Translational Modification |  |  | ✓ |
|  | Protein Synthesis |  | ✓ |  |
|  | Protein Trafficking |  | ✓ |  |
|  | RNA Trafficking | ✓ |  |  |
|  | Small Molecule Biochemistry |  |  | ✓ |
| **Physiological System Development and Function** | Cardiovascular System Development and Function |  | ✓ |  |
|  | Cell-mediated Immune Response |  | ✓ |  |
|  | Hematological System Development and Function |  | ✓ |  |
|  | Immune Cell Trafficking |  | ✓ |  |
|  | Nervous System Development and Function |  | ✓ |  |
|  | Organ Development |  | ✓ |  |
|  | Skeletal and Muscular System Development and Function |  |  | ✓ |
|  | Tissue Development |  | ✓ |  |
|  | Tumor Morphology |  | ✓ |  |
|  | Visual System Development and Function |  | ✓ |  |
