# Supplementary material for: Hybrid Models Identified a 12-Gene Signature for Lung Cancer Prognosis and Chemoresponse Prediction
Source: PLoS One. 2010 Aug 17;5(8):e12222. doi: 10.1371/journal.pone.0012222 (PMC2923187; doi:10.1371/journal.pone.0012222)
Supplement: Table S6 — 14 published lung cancer gene signatures evaluated in GSEA. (0.05 MB DOC) [file pone.0012222.s006.doc]

| **Signature Name (GSEA)** | **First Author** | **Publication PubMed ID** | **No. of Signature Genes/Probes** | **No. of Genes matched in GSEA (By gene symbol)** |
| --- | --- | --- | --- | --- |
| Beer_50g | Beer, DG | PMID:12118244 | 50 | 45 |
| Bhattacharjee_150g | Bhattacharjee, A | PMID:11707567 | 150 | 130 |
| Boutros_6g | Boutros, PC | PMID:19196983 | 6 | 6 |
| Chen_5g | Chen, HY | PMID:17202451 | 5 | 5 |
| Guo_35g | Guo, L | PMID:16740756 | 35 | 34 |
| Lau_3g | Lau, SK | PMID:18065728 | 3 | 3 |
| Lu_64g | Lu, Y | PMID:17194181 | 64 | 62 |
| Potti_133g | Potti, A | PMID:16899777 | 133 | 129 |
| Raponi_50g | Raponi, M | PMID:16885343 | 50 | 44 |
| Shedden_MA | Shedden, K | PMID:18641660 | 13830 | 8319 |
| Shedden_MB | Shedden, K | PMID:18641660 | 52 | 50 |
| Shedden_MC | Shedden, K | PMID:18641660 | 26 | 23 |
| Shedden_MD | Shedden, K | PMID:18641660 | 42 | 34 |
| Shedden_MH | Shedden, K | PMID:18641660 | 313 | 244 |
