# Supplementary material for: Hybrid Models Identified a 12-Gene Signature for Lung Cancer Prognosis and Chemoresponse Prediction
Source: PLoS One. 2010 Aug 17;5(8):e12222. doi: 10.1371/journal.pone.0012222 (PMC2923187; doi:10.1371/journal.pone.0012222)
Supplement: Table S7 — Summary of gene selection and classification methods of molecular classifiers compared in Fig. 5. Gene signatures A-N were reported in (Shedden et al, 2008). (0.05 MB DOC) [file pone.0012222.s007.doc]

| **Molecular Classifier*** | **Number of signature genes** | **Gene selection method(s)** | **Classification method(s)** |
| --- | --- | --- | --- |
| Shedden A | ~ 9591 Genes | Clustering analysis | Ridged Cox proportional hazard model |
| Shedden C | 23 Genes | SAM, Maximizing Chi-Square analysis (MCA, univariate Cox model and k-mean clustering) | Binary Tree-Structured Vector Quantization (BTSVQ) |
| Shedden D | 37 Genes | SAM, Maximizing Chi-Square analysis (MCA, univariate Cox model and k-mean clustering) | Binary Tree-Structured Vector Quantization (BTSVQ) |
| Shedden E | 1 Gene | Gene Expression Fold Change | Post-hoc split of expression of one gene |
| Shedden F | 42 Genes | Univariate Cox Model | Principal Components and Cox Model |
| Shedden G | 38 Genes | Univariate Cox Model | Principal Components and Cox Model |
| Shedden H | 252 Genes | Scoring and filtering on set of mitosis genes | Majority vote |
| Shedden J | 5 Genes | Univariate Cox model (Chen et al, NEJM 07) | Ridged Cox proportional hazard model |
| Shedden K | 16 Genes | Univariate Cox model (Chen et al, NEJM 07) | Ridged Cox proportional hazard model |
| Shedden L | 9 Genes  (from 80 Genes) | Principal Components (Potti et al, NEJM 06) | Ridged Cox proportional hazard model |
| Shedden M | 45 Genes  (from 80 Genes) | Principal Components (Potti et al, NEJM 06) | Ridged Cox proportional hazard model |
| Shedden N | 80 Genes | Principal Components (Potti et al, NEJM 06) | Ridged Cox proportional hazard model |
| 12-gene | 12 Genes | t-test, SAM, RELIEFF | Naïve Bayes |

*Gene signatures A-H were identified in (Shedden et al, 2008). Gene signatures J and K were identified in (Chen et al, 2007). Gene signatures L, M, and N were identified in (Potti et al, 2006).
