# Supplementary material for: Hybrid Models Identified a 12-Gene Signature for Lung Cancer Prognosis and Chemoresponse Prediction
Source: PLoS One. 2010 Aug 17;5(8):e12222. doi: 10.1371/journal.pone.0012222 (PMC2923187; doi:10.1371/journal.pone.0012222)
Supplement: Table S8 — Machine learning algorithm and genes used in chemoresponse prediction using 12-gene signature. (0.05 MB DOC) [file pone.0012222.s008.doc]

| **Anti-cancer Agent** | **Machine learning algorithm** | **Genes Selected** | **Resistant lung cancer cell lines** | **Sensitive lung cancer cell lines** |
| --- | --- | --- | --- | --- |
| **Carboplatin** | RBF Network (seed = 2) | ATP6V0D1  CCDC99  FAM164A  LMF1  PDPK1  PKLR  SCLY  SMPD1  STK24  XPO1 | LC:EKVX  LC:NCI_H322M | LC:NCI_H460  LC:NCI_H522  (LC:NCI_H23 not included due to missing values) |
| **Paclitaxel** | IBK (k=3) | CCDC99  DLC1  LMF1  PKLR  SMPD1  XPO1  ZAK | LC:HOP_92  LC_EKVX | LC:NCI_H460  LC:NCI_H522 |
| **Cisplatin** | Decorate (PART as base learner) | ATP6V0D1  CCDC99  FAM164A  LMF1 | LC:NCI_H226  LC:EKVX  LC:NCI_H322M | LC:HOP_62  LC:NCI_H460  (LC:NCI_H23 not included due to missing values) |
| **Etoposide** | AdaBoostM1 (seed = 2, Random Tree as base learner) | CCDC99  LMF1  SCLY  STK24  XPO1 | LC:EKVX  LC:NCI_H322M | LC:HOP_62  LC:NIC_H460 |
| **Erlotinib** | RBF Network | DLC1  LMF1  XPO1  SMPD1  STK24  PDPK1  ZAK  PKLR  CCDC99 | LC:NCI_H226  (LC:NCI_H23 not included due to missing values) | LC:EKVX  LC:NCI_H322M  LC:NCI_H522 |
| **Gefitinib** | Multilayer Perceptron (seed=2, learning rate=0.4) | ATP6V0D1  SMPD1  XPO1  PKLR  STK24  SCLY | LC:A549  LC:HOP_62  LC:HOP_92  LC:NCI_H226  (LC:NCI_H23 not included due to missing values) | LC:EKVX  LC:NCI_H322M |
