# Supplementary material for: Hybrid Models Identified a 12-Gene Signature for Lung Cancer Prognosis and Chemoresponse Prediction
Source: PLoS One. 2010 Aug 17;5(8):e12222. doi: 10.1371/journal.pone.0012222 (PMC2923187; doi:10.1371/journal.pone.0012222)
Supplement: Table S9 — Sensitivity and specificity of the 12-, 15- and 16-gene prognostic models. (0.05 MB DOC) [file pone.0012222.s009.doc]

|  | **Sensitivity (% of correctly predicted high-risk patients)** | | | |  | **Specificity (% of correctly predicted low-risk patients)** | | | |
| --- | --- | --- | --- | --- | --- | --- | --- | --- | --- |
|  | ***n*** | **12-gene** | **15-gene** | **16-gene** |  | ***n*** | **12-gene** | **15-gene** | **16-gene** |
| ***3-year survival as the cutoff (high-risk: death within 3-y; low-risk: alive after 3-y)*** | | | | | | | | | |
| **UM & HLM** | 95 | 73.65 | 76.84 | 47.37 |  | 152 | 59.21 | 64.47 | 87.50 |
| **MSK** | 23 | 86.96 | 82.61 | 60.87 |  | 71 | 57.75 | 50.70 | 70.42 |
| **DFCI** | 22 | 68.18 | 86.36 | 54.55 |  | 55 | 76.36 | 47.27 | 81.82 |
| ***5-year survival as the cutoff (high-risk: death within 5-y; low-risk: alive after 5-y)*** | | | | | | | | | |
| **UM & HLM** | 125 | 72.80 | 72.80 | 44.80 |  | 104 | 66.35 | 69.23 | 93.27 |
| **MSK** | 34 | 70.59 | 67.65 | 50.00 |  | 31 | 48.39 | 41.94 | 67.74 |
| **DFCI** | 28 | 64.29 | 78.57 | 50.00 |  | 36 | 77.78 | 47.22 | 86.11 |
| ***2.5-year and 5-year survival as the high- and low-risk cutoffs (high-risk: death within 2.5-y; low-risk: alive after 5-y)*** | | | | | | | | | |
| **UM & HLM** | 84 | 75.00 | 77.38 | 48.81 |  | 104 | 66.35 | 69.23 | 93.27 |
| **MSK** | 21 | 95.24 | 85.71 | 66.67 |  | 31 | 48.39 | 41.94 | 67.74 |
| **DFCI** | 20 | 70.00 | 85.00 | 55.00 |  | 36 | 77.78 | 47.22 | 86.11 |
