# Supplementary figures and images for: Hybrid Models Identified a 12-Gene Signature for Lung Cancer Prognosis and Chemoresponse Prediction
Source: PLoS One. 2010 Aug 17;5(8):e12222. doi: 10.1371/journal.pone.0012222 (PMC2923187; doi:10.1371/journal.pone.0012222)

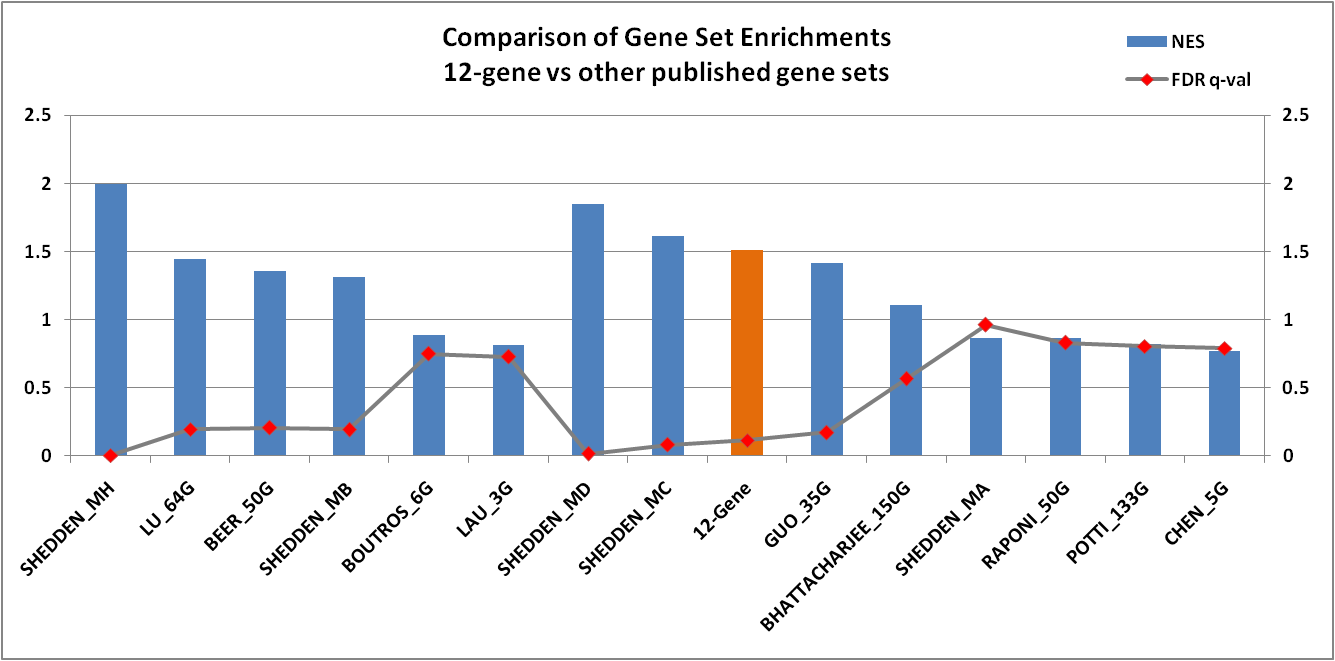

Supplement: Figure S1 — Gene set enrichment analysis of the 12-gene signature along with 14 published gene signatures for NSCLC. A summary of the 14 gene signatures analyzed is listed in Table S6. (0.10 MB TIF) [file pone.0012222.s010.tif]

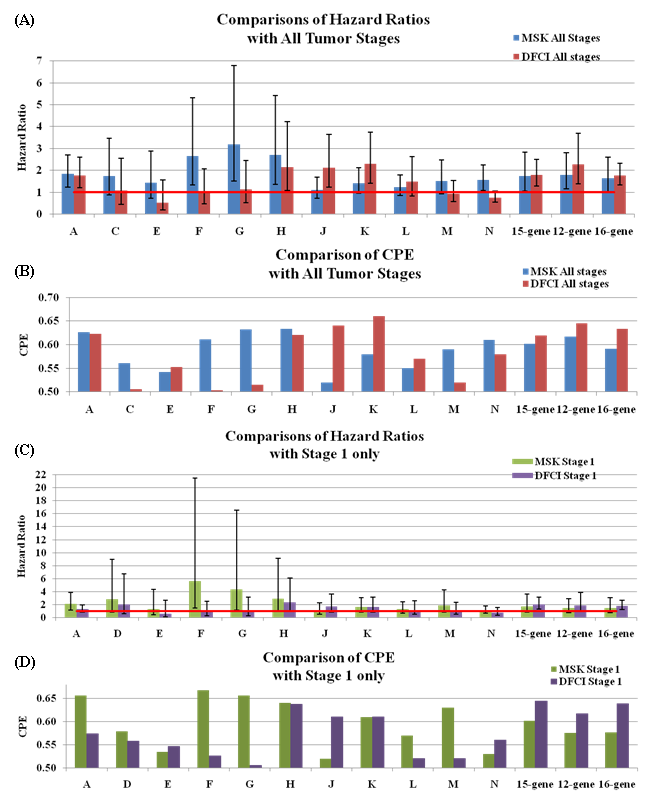

Supplement: Figure S2 — Evaluation of the 15-gene, 12-gene, and 16-gene prognostic models with molecular prognostic models presented by Shedden et al (2008). Hazard ratio (A, C) and concordance probability estimate (CPE) (B, D) were compared on patients in all stages (A, B) and stage I (C, D) of lung cancer. Error bars in (A) and (C) represent 95% confidence interval of hazard ratio. (0.15 MB TIF) [file pone.0012222.s011.tif]

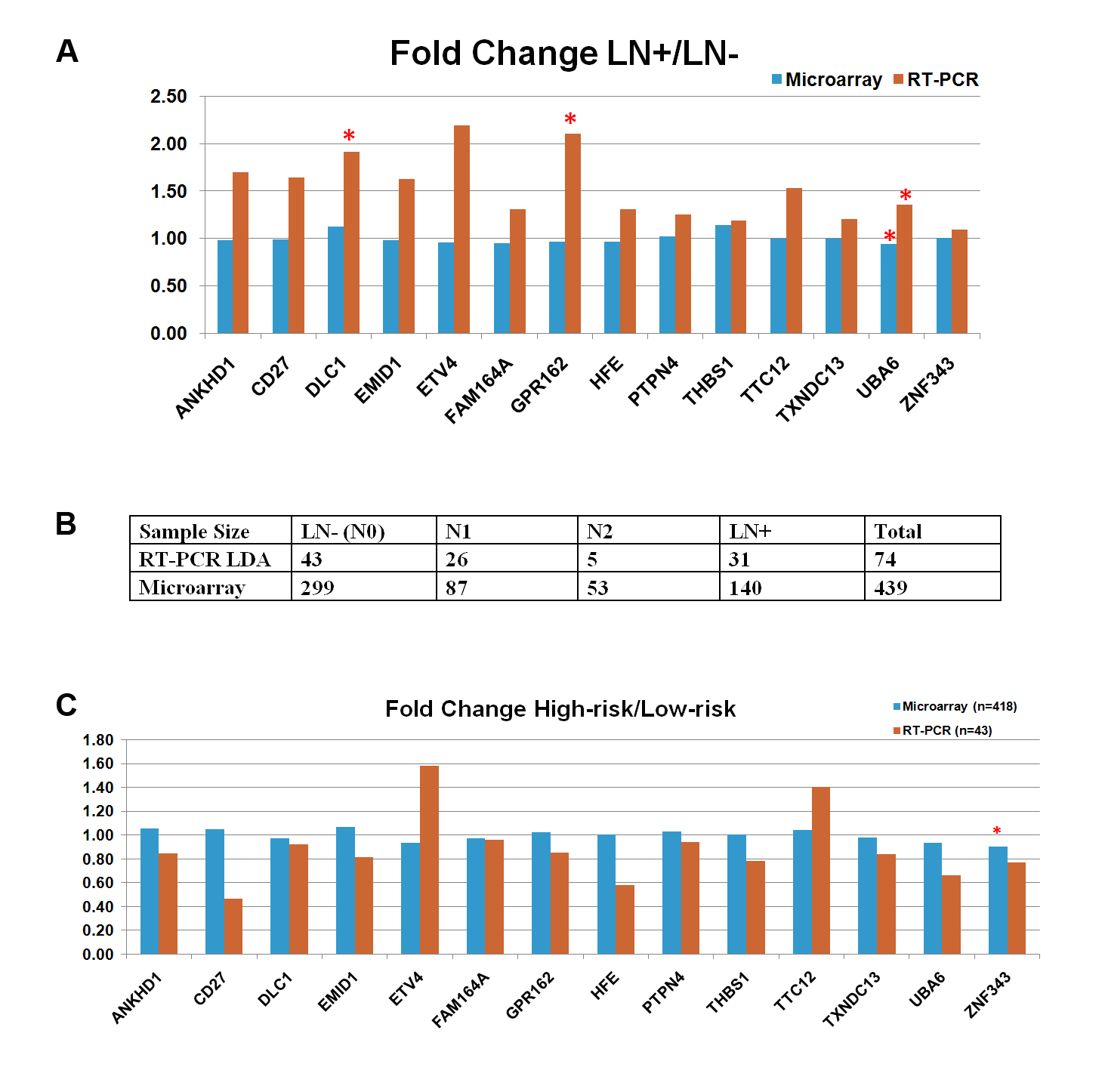

Supplement: Figure S3 — Comparison of gene expression patterns of the 15-gene signature measured with DNA microarray and RT-PCR microfluidic low density arrays (LDA). Gene expression fold change in lymph node positive (LN+) patients vs. lymph node negative (LN−) patients was compared (A). Samples included in the fold change comparison are summarized in (B). On patient with follow-up information, gene expression fold change in high-risk patients vs. low-risk patients at 3-year period after surgery was also compared (C). The RT-PCR data were normalized with POLR2A in a sample-wise manner. DNA microarray data were obtained from Shedden et al (2008). Red asterisk (*) above the bar indicates the gene was differentially expressed t-test (P<0.05). (0.22 MB TIF) [file pone.0012222.s012.tif]
